# Metagenomes and metatranscriptomes shed new light on the microbial-mediated sulfur cycle in a Siberian soda lake

**DOI:** 10.1186/s12915-019-0688-7

**Published:** 2019-08-22

**Authors:** Charlotte D. Vavourakis, Maliheh Mehrshad, Cherel Balkema, Rutger van Hall, Adrian-Ştefan Andrei, Rohit Ghai, Dimitry Y. Sorokin, Gerard Muyzer

**Affiliations:** 1 Microbial Systems Ecology, Department of Freshwater and Marine Microbiology, Institute for Biodiversity and Ecosystem Dynamics, University of Amsterdam, P.O. Box 94240, 1090 GE Amsterdam, the Netherlands; 2 Department of Aquatic Microbial Ecology, Institute of Hydrobiology, Biology Centre of the Academy of Sciences of the Czech Republic, České Budějovice, Czech Republic; 3 Department of Ecosystem & Landscape Dynamics, Institute for Biodiversity and Ecosystem Dynamics, University of Amsterdam, Amsterdam, the Netherlands; 4 Winogradsky Institute of Microbiology, Research Centre of Biotechnology, Russian Academy of Sciences, Moscow, Russian Federation; 5 Department of Biotechnology, Environmental Biotechnology, Delft University of Technology, Delft, the Netherlands

**Keywords:** Soda lake, Haloalkaliphiles, Metagenomics, Metatranscriptomics, Thiosulfate, Tetrathionate, Polysulfide, *Woesearchaeota*, *Gemmatimonadetes*, Nitrogen fixation

## Abstract

**Background:**

The planetary sulfur cycle is a complex web of chemical reactions that can be microbial-mediated or can occur spontaneously in the environment, depending on the temperature and pH. Inorganic sulfur compounds can serve as energy sources for specialized prokaryotes and are important substrates for microbial growth in general. Here, we investigate dissimilatory sulfur cycling in the brine and sediments of a southwestern Siberian soda lake characterized by an extremely high pH and salinity, combining meta-omics analyses of its uniquely adapted highly diverse prokaryote communities with biogeochemical profiling to identify key microbial players and expand our understanding of sulfur cycling under haloalkaline conditions.

**Results:**

Peak microbial activity was found in the top 4 cm of the sediments, a layer with a steep drop in oxygen concentration and redox potential. The majority of sulfur was present as sulfate or iron sulfide. Thiosulfate was readily oxidized by microbes in the presence of oxygen, but oxidation was partially inhibited by light. We obtained 1032 metagenome-assembled genomes, including novel population genomes of characterized colorless sulfur-oxidizing bacteria (SOB), anoxygenic purple sulfur bacteria, heterotrophic SOB, and highly active lithoautotrophic sulfate reducers. Surprisingly, we discovered the potential for nitrogen fixation in a new genus of colorless SOB, carbon fixation in a new species of phototrophic *Gemmatimonadetes*, and elemental sulfur/sulfite reduction in the “*Candidatus* Woesearchaeota.” Polysulfide/thiosulfate and tetrathionate reductases were actively transcribed by various (facultative) anaerobes.

**Conclusions:**

The recovery of over 200 genomes that encoded enzymes capable of catalyzing key reactions in the inorganic sulfur cycle indicates complete cycling between sulfate and sulfide at moderately hypersaline and extreme alkaline conditions. Our results suggest that more taxonomic groups are involved in sulfur dissimilation than previously assumed.

**Electronic supplementary material:**

The online version of this article (10.1186/s12915-019-0688-7) contains supplementary material, which is available to authorized users.

## Background

Since the dawn of unicellular life, the dissimilation of sulfur compounds by specialized prokaryotes forms an important part of the planetary biogeochemical cycle of sulfur [1–3]. For instance, microbial sulfide oxidation lies at the base of the food chain in the ecosystems associated with hydrothermal vents in the deep sea [4], and sulfate reducers are the primary degraders of organic material in the seabed where oxygen is depleted [5]. A variety of stable intermediary inorganic sulfur compounds are biologically available in between the end members of the sulfur cycle, free dissolved sulfide (ƩH_2_S = H_2_S + HS^−^ + S^2−^), and sulfate (SO_4_^2−^). In addition, different temperature- and pH-dependent abiotic conversions occur; sulfur can react with other elements and can be cycled in between organic and inorganic compounds, rendering the biogeochemical cycle of sulfur quite complex.

Hypersaline soda lakes are evaporative terrestrial lakes with total salt concentrations above 50 g L^−1^ and a stable high pH roughly around 10 [6, 7]. These unique environmental conditions form a strong selective force and microbes adapted to these double extremes are called “haloalkaliphiles.” Driven by high sulfate concentrations and primary productivity in the brines, the inorganic sulfur cycle is one of the most active cycles occurring in soda lakes [7–11]. At high pH, ƩH_2_S occur mainly in the deprotonated hydrosulfide form (HS^−^). Sulfide oxidation and sulfide production by reduction of sulfur compounds and the degradation of organic sulfur, i.e., sulfidogenesis, are therefore not hampered by the buildup of toxic H_2_S, as is the case in environments with acidic or neutral pH [8]. Due to the high alkalinity, increased chemical stability of polysulfides (S_*n*_^2−^) in anoxic sediments also results in relatively higher rates of sulfur-polysulfide respiration compared to dissimilatory sulfate reduction. At moderate salinities (~ 50–250 g L^−1^ total salt), a complete sulfur cycle between ƩH_2_S and SO_4_^2−^ is proposed to occur, whereas at salt-saturating conditions, the cycle is likely short-circuited by the presence of sulfur intermediates such as elemental sulfur (S^0^), polysulfides (S_*n*_^2−^), and thiosulfate (S_2_O_3_^2−^) [8].

Except for phototrophic green sulfur bacteria (family *Chlorobiaceae*), haloalkaliphilic representatives have been isolated for all known functional groups involved in the dissimilatory inorganic sulfur cycle [7–10]. Purple sulfur bacteria from the genera *Halorhodospira* and *Ectothiorhodospira* (class *Gammaproteobacteria*) that use light energy and some reduced sulfur compounds for anoxygenic photosynthesis are also commonly found in other high-salinity environments with neutral pH [12]. However, most genera appear to be unique for soda lakes and industrial haloalkaline environments [10], such as the chemolithoautotrophic sulfur-oxidizing bacteria (SOB) from the genus *Thioalkalivibrio* (class *Gammaproteobacteria*) that can use diverse reduced sulfur compounds as electron donors and bacteriochlorophyll-*a* containing lithoheterotrophic SOB from the genera *Roseinatronobacter* and *Rhodobaca* (class *Alphaproteobacteria*) that can use sulfur compounds as an additional energy source. The known genera of the soda lake sulfidogens, which perform sulfate reduction, elemental sulfur/polysulfide or thiosulfate reduction, and disproportionation, all appear to be obligate haloalkaliphiles [7]. Recently, it has been discovered that also the members of the extremely haloalkaliphilic *Euryarchaeota* can participate in dissimilatory sulfur respiration in anaerobic sediments of hypersaline soda lakes [13].

Despite the broad interest in the global biogeochemical cycle of sulfur, the genetic makeup of the microbes involved especially in the oxidative part of the cycle and those that disproportionate intermediary compounds is not yet fully understood [14–18]. Both phototrophic and chemotrophic sulfur oxidizers share the same enzymes for sulfur transformations [19, 20]. The best described pathway of thiosulfate oxidation to SO_4_^2−^ occurs through the “Sox” enzyme system, with SoxAX, SoxYZ, SoxB, and SoxCD as the essential components [21]. Intermediary storage of zero-valent sulfur in sulfur globules occurs only under suboptimal environmental conditions in organisms that lack SoxCD [22]. Further oxidation of the zero-valent sulfur can involve several other enzymes, such as a reversed dissimilatory sulfite reductase (rDSR), sulfate adenylyltransferase together with adenylyl-sulfate reductase (Sat/Apr), and sulfite dehydrogenases (SOR/SOE), but uncertainties remain as many SOB lack the rDSR [14]. Two alternative pathways for thiosulfate oxidation have been described that involve the formation of tetrathionate (S_4_O_6_^2−^) which include the quinone-interacting doxAD/TETH system described in the acidophilic elemental sulfur-oxidizing archaeon *Acidianus ambivalens* [23] or the cytochrome *c*-dependent *tsdAB*-encoded thiosulfate dehydrogenase in *Allochromatium vinosum* [24, 25]*.*

Several sulfur intermediates can be cycled through combined microbial-chemical processes in an intraspecies sulfur cycle. For instance, certain heterotrophic members of the *Gammaproteobacteria* oxidize S_2_O_3_^2−^ to S_4_O_6_^2−^ in soda lakes. The S_4_O_6_^2−^ is released by the cells and can act as an oxidant on ƩH_2_S in the environment leading to the formation of S^0^ and the regeneration of S_2_O_3_^2−^ [26]. Intraspecies sulfur cycling is also proposed to occur with bacterial S^0^ and S_2_O_3_^2−^ reduction catalyzed by polysulfide/thiosulfate reductases (*psr/phs* genes) in several neutrophilic model organisms [27–29]. Polysulfides formed by the reaction of S^0^ and HS^−^ are reduced in the periplasm of *Wolinella succinogenes* (class *Epsilonproteobacteria*) to HS^−^ and S_*n*-1_^2−^, after which the HS^−^ diffuses out of the cell and reacts again with S^0^ to form S_*n*_^2−^ [28]. Recently, a *psrA/phsA* orthologous gene was also identified in the genome of the haloalkaliphilic sulfidogen *Desulfurivibrio alkaliphilus* AHT2^T^ [30], an organism that can perform elemental sulfur (polysulfide) disproportionation [18].

While more physiological studies of novel isolates and enzyme characterizations are necessary to close the knowledge gaps that remain in the sulfur biogeochemical cycle, culture-independent methods in combination with in situ rate measurements help to pinpoint where significant gaps still exist. Direct sequencing of environmental DNA or RNA, i.e., metagenomics and metatranscriptomics, which avoid the bottleneck of cultivation, has the potential to characterize the genetic capabilities and regulation of gene expression in novel, uncultivated organisms. Previous meta-omics studies on hypersaline soda lakes have targeted the sulfur cycle mainly through the detection of functional marker genes or their transcripts rather than focusing on the reconstruction of metagenome-assembled genomes (MAGs) [31–33] or have focused on the MAGs of only the most abundant uncultured microbes present in soda lake brines or sediments [34, 35].

Here, we investigated the inorganic sulfur cycle in Cock Soda Lake (Kulunda Steppe, southwestern Siberia, Russia). We chose this lake specifically because in contrast to the smaller lakes in its close vicinity, it has a relatively stable water regime with a moderately hypersaline brine (50–120 g L^−1^ salt, pH 10) where a complete sulfur cycle could occur. We identified for 1032 newly recovered MAGs from the brine and sediments which prokaryotes encode marker genes for oxidative and reductive branches of the dissimilatory sulfur cycle. Further, transcription by putative sulfur-cycling prokaryotes was investigated using both sequencing of the RNA from the metatranscriptome and sequencing amplicons of 16S rRNA gene transcripts from samples of the top sediment layer.

## Results

### Biogeochemical profile of Cock Soda Lake

**Fig. 1:**
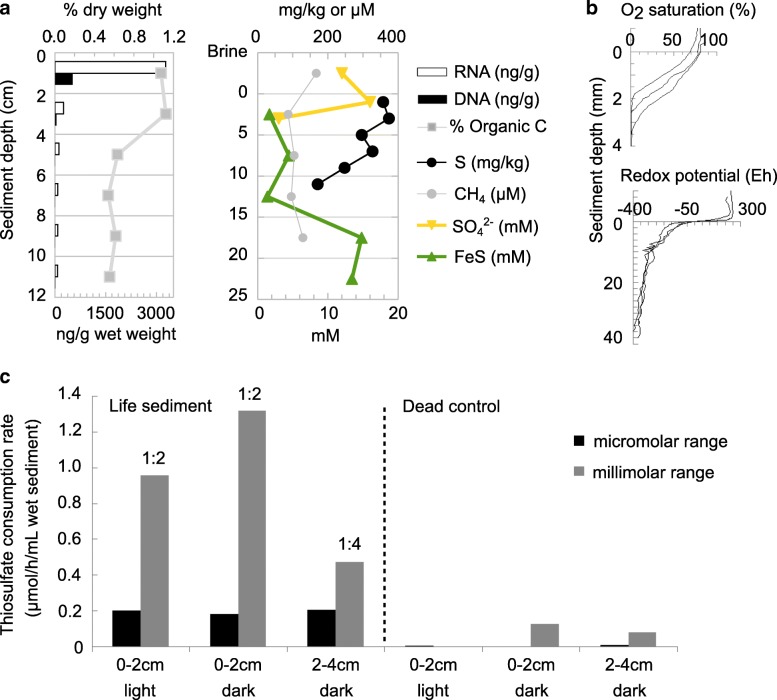
Water and sediment biogeochemical depth profiles from Cock Soda Lake. **a** Nucleic acid extraction efficiencies, total organic carbon, total sulfur measured by ICP-MS (S), and methane and inorganic sulfur compound concentrations. **b** Oxygen saturation and oxidation-reduction potential (redox) microsensor profiles obtained for three sediment columns. Each line is the average measured on three different spots. **c** Rates of thiosulfate oxidation determined for different sediment layers of Cock Soda Lake in the dark or light after 24-h incubation under oxic conditions with different amounts of thiosulfate. Oxidation is microbial mediated and proceeds considerably higher in the millimolar range. The fastest rates were obtained mediated by the microbiota from the top 2 cm under dark conditions

The total salinity of the Cock Soda Lake brine in July 2016 was 55 g L^−1^, and the soluble carbonate alkalinity was 0.62 M. Based on the measured chloride ion concentrations and inorganic carbon, we inferred that the total salinity decreased only slightly in the pore water of the sediments below 2 cm depth (Additional file 1: Figure S1). The pH of the brine and the pore water of the top 2 cm sediment layer was 9.9 (Additional file 2: Figure S2). The brine was green in color, teeming with live brine shrimps and strongly mixed by wind. The larvae of the soda fly *Ephydra hyans* [36] actively perturbed the top layer of the sediment, which was covered by a thin fluffy green mat. Organic carbon contents in the surface 4 cm were > 1% and decreased to approximately 0.5% in the sediments below 4 cm (Fig. 1a). Nucleic acid isolation yields were 13–274× higher from samples taken from the top 2 and 5 cm sediment layers compared to deeper layers. The top 12 cm of the sediment matrix consisted of silty sand with a mean grain size of 123 μm and a bimodal distribution of mostly very fine or medium sand grains and 20% silt (i.e., particles < 63 μm; Additional file 3: Table S1). The top 12 cm sediment fraction was rich in iron (~ 6–8 g/kg), phosphorus (~ 130–200 mg/kg), and manganese (~ 120–190 mg/kg) (Additional file 3: Table S2). In the brine and the top 2 cm of the sediments, most of the measured sulfur compounds were present as sulfate ions (Fig. 1a). Deeper in the sediments, total sulfur content (ICP-OES) decreased and was mainly in the form of acid-volatile iron sulfides (FeS). Traces of S_2_O_3_^2−^ were measured only in the surface sediment (~ 40 μM and ~ 20 μM in the 0–2-cm and 2–4-cm layer, respectively) from which enough pore water could be extracted. Nitrate concentrations in the pore water could only be quantified in the 2–4-cm sediment layer (~ 100 μM; Additional file 1: Figure S1a). No free hydrogen sulfide (HS^−^) could be detected. The highest methane concentration was measured in the brine, but it was detectable throughout the whole sediment column as well. The redox potential dropped steeply in the first centimeter of sediments, a layer with a distinct brown coloration, and gradually decreased to approximately − 400 Eh at 3.5-cm sediment depth (Fig. 1b). The dissolved oxygen concentration in the pore water dropped below detection in the top 2–4 mm of sediments.

The rates of microbial thiosulfate consumption in the 0–2-cm and 2–4-cm sediment layers were comparable (Fig. 1c). Two- to sevenfold higher rates of S_2_O_3_^2−^ consumption were observed at millimolar compared to micromolar concentrations. For the experiments in the millimolar range, S_2_O_3_^2−^ was completely oxidized to SO_4_^2−^ (1:2 M ratios) in the top 2-cm sediment layer both in the light and the dark, but the relatively higher rates obtained under dark conditions suggest partial inhibition by light. In the 2–4-cm sediment layer, the measured S_2_O_3_^2−^ to SO_4_^2−^ ratio shifted to 1:4, because more reduced sulfur compounds (most probably FeS) were additionally oxidized upon oxygen exposure.

### Prokaryotic community profiles in the brine and top 25 cm of sediments

**Fig. 2:**
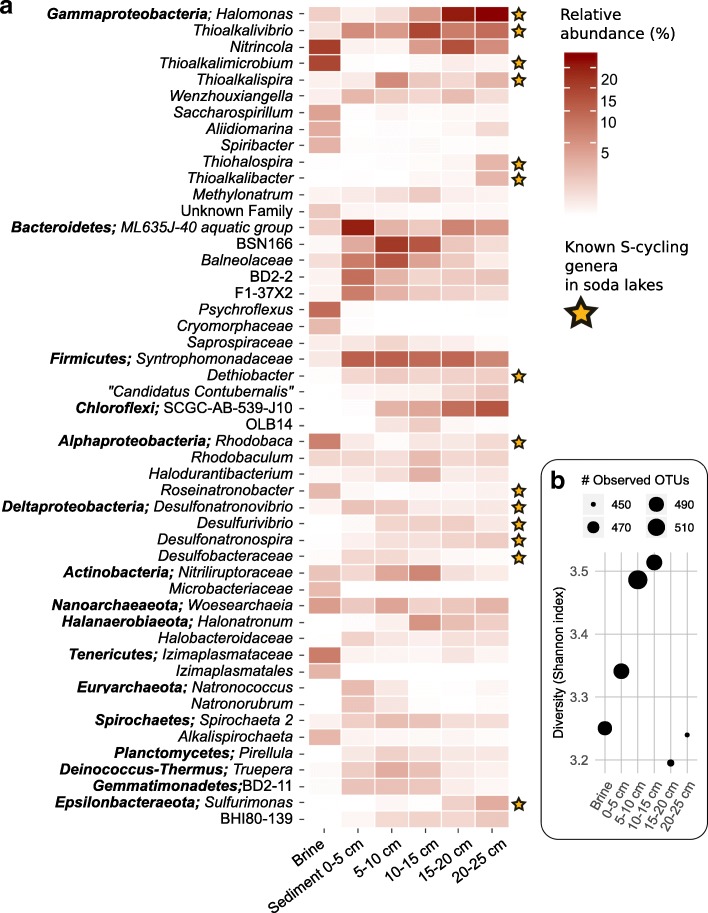
**a** Average relative abundance of the top 50 abundant genera or OTUs in Cock Soda Lake identified by 16S rRNA gene amplicon sequencing. The minimum relative abundance shown is 0.1% (white). The yellow stars indicate the genera from which soda lake isolates were previously characterized that have the ability to transform inorganic sulfur compounds. **b** Richness and diversity of the prokaryote communities in the soda lake brine and at different sediment depths

The brine and sediment prokaryotic community profiles obtained by 16S rRNA gene amplicon sequencing were very different (Fig. 2a). Community diversity at the level of genus increased from the brine down to a depth of 15 cm in the sediments. A steep drop in the total number of observed OTUs (~ richness) and Shannon diversity was found in the 15–25-cm sediment layer (Fig. 2b), which coincided with a steep increase in the amount of FeS at this depth (Fig. 1a). The most abundant genus in the brine was *Nitrincola* (*Gammaproteobacteria*), which contains haloalkaliphilic, facultative anaerobic isolates capable of nitrate reduction [37] (Fig. 2a). The 50 most abundant genera amounted to a total relative abundance of ~ 80–90% of the prokaryotic community in every sediment layer. Those OTUs assigned to the genera known to be involved in dissimilatory cycling of inorganic sulfur compounds [7] were relatively more abundant in the sediment. Only the SOB genera *Thioalkalimicrobium* (reclassified recently into *Thiomicrospira* [38], *Gammaproteobacteria*), *Rhodobaca*, and *Roseinatronobacter* (*Alphaproteobacteria*) were predominantly found in the brine (~ 15, 7, and 2%, respectively). Chemolithoautotrophic SOB from the genus *Thioalkalivibrio* were highly abundant at every sediment depth (~ 5–15%), while the genera *Thiohalospira*, *Thioalkalibacter*, *Thioalkalispira* (*Gammaproteobacteria*), and *Sulfurimonas* (*Epsilonbactereota*) were relatively more abundant deeper in the sediment. The most abundant genus at 15–25-cm sediment depth (~ 21–23%) was *Halomonas*, from which haloalkaliphilic isolates can oxidize thiosulfate to tetrathionate [26]. The second most abundant genus in the deepest sediment layers was an uncultured group SCGC-AB-539-J10 within the *Chloroflexi* (~ 9–12%, *Dehalococcoidia*). The members of this group are also found in marine subsurface sediments, but their exact ecological role remains to be determined [39]. Lithoautotrophic sulfate-reducing bacteria (SRB) from the genera *Desulfonatronovibrio* and *Desulfonatronospira* (*Deltaproteobacteria*) and sulfur/thiosulfate reducers capable of lithoauthotrophic polysulfide disproportionation from the known genera *Desulfurivibrio* (*Deltaproteobacteria*) and *Dethiobacter* (*Firmicutes*) constituted together a significant portion of the total community in every sediment layer (~ 3–4%).

### High-throughput genome recovery from the brine and top layer of sediments

**Fig. 3:**
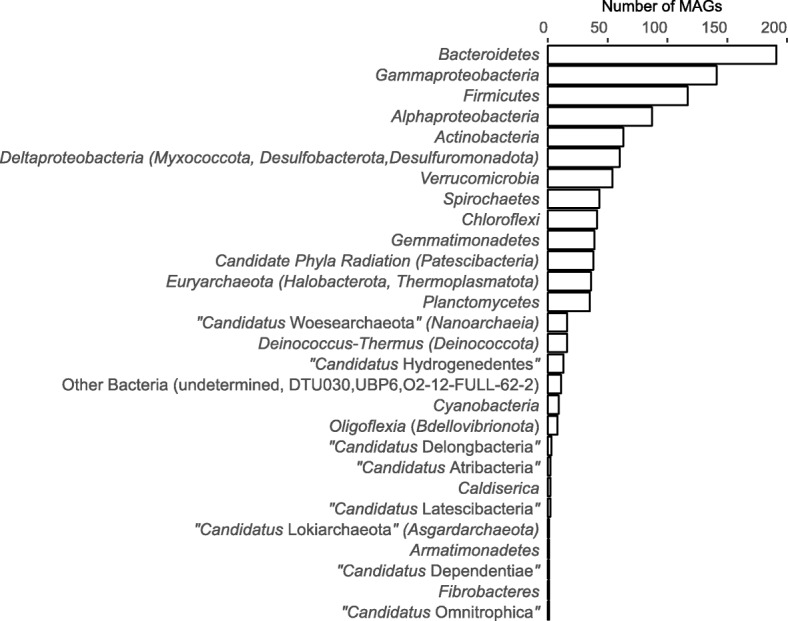
Phylogeny (phylum/class level) of the 1032 novel metagenome-assembled genomes (MAGs) obtained from Cock Soda Lake in this study. In the brackets are the phylum- or class-level “GTDB” taxonomic names indicated according to [89]

We reconstructed in total 1032 novel MAGs from the brine and top layers (0–2 and 0–5 cm) of the sediments, among which 232 were high-quality drafts (CheckM-completeness ≥ 90%, CheckM-contamination < 5%) (Additional file 4: Dataset 1) [40]. The MAGs were assigned to at least 28 different phyla (Fig. 3). About half of the MAGs were classified as *Proteobacteria* (288), *Bacteroidetes* (191), and *Firmicutes* (117), which is in agreement with the dominant phyla found in the 16S rRNA gene amplicon sequencing datasets (Fig. 2). Within the *Bacteroidetes* and *Firmicutes*, most MAGs were assigned the same taxonomy (Additional file 4: Dataset 1) as the major dominant OTUs in the top 5 cm of sediments (Fig. 2), namely the ML635J-40 aquatic group (SILVA database) and the *Syntrophomonadaceae*, respectively, putative haloalkaliphilic groups commonly detected in soda lakes [35]. The latter family members might be reversed acetogens, able to oxidize acetate in syntrophy with methanogens or SRB, or they might be hydrogenotrophic acetogens [35, 41]. The most abundant 16S rRNA gene transcripts were assigned to *Nodosilinea* (relative abundance ~ 17%; a genus of haloalkaliphilic, filamentous benthic *Cyanobacteria* [42, 43]) in the 0–2-cm layer of sediments and to *Nitriliruptoraceae* (~ 17%; a family of putative nitrile-hydrolyzing *Actinobacteria* [44]) in the 2–4-cm layer (Additional file 5: Figure S3), groups from which we recovered 1 and 38 different MAGs, respectively (Additional file 4: Dataset 1).

Six MAGs could not be classified to any known phylum using the Genome Taxonomy Database (GTDB) (“Other Bacteria, undetermined”; Fig. 3). Based on the phylogeny of 16S rRNA genes (Additional file 4: Dataset 1) and conserved ribosomal proteins (Additional file 6: Dataset 2), four out of six unclassified MAGs (bin. CSSed162cmB.61, bin. CSSed165cm.362, bin. CSSed165cm.369, and bin. CSSed165cm.452) were closely affiliated with the bacterial candidate division BRC1 and the remaining two MAGS (bin. CSSed165cm.289 and bin. CSSed165cm.465) were distantly related to the uncultured group LD1-PA32 (*Chlamydiae*).

### Metagenome-assembled genomes and transcriptional activity of dissimilatory sulfur prokaryotes

**Fig. 4:**
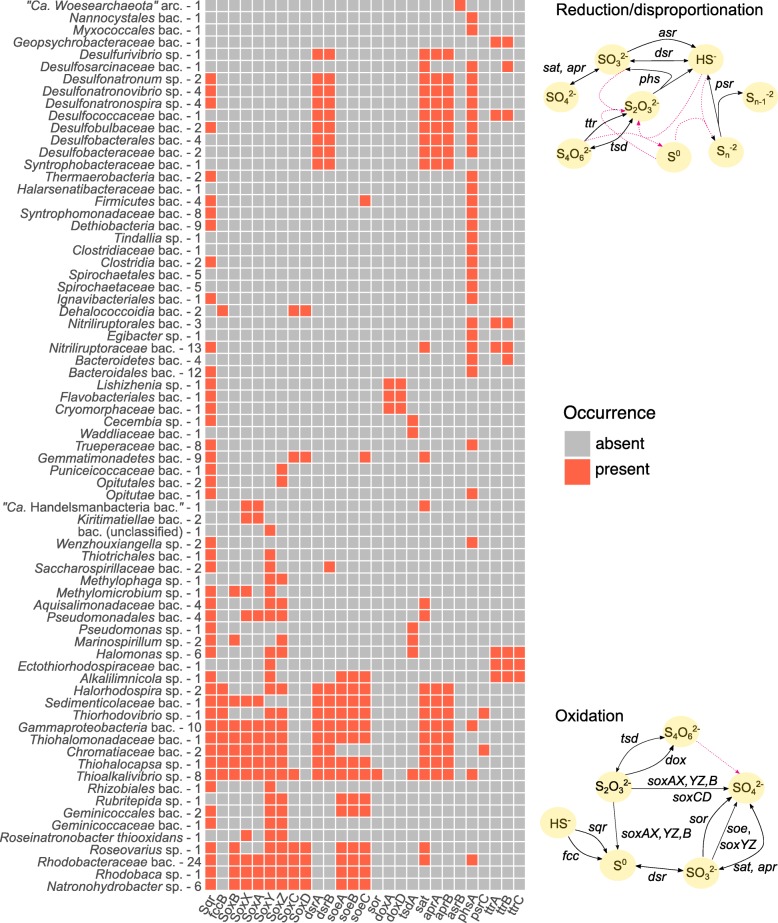
Overview pathways involved in dissimilatory inorganic sulfur compound cycling encoded by the selected MAGs. The presence/absence of functional marker genes in the MAGs is given by the color scheme. The genomes were grouped by phylogeny, and the number of species representatives assessed for each group is given after its taxonomic name. The involvement of each gene in specific pathways is indicated in the diagrams. Full arrows indicate the enzymatic reactions for which genes were found encoded in the metagenomes. The dotted, pink arrows show possible chemical reactions

Among the 1032 new MAGs and 401 MAGs obtained previously from the Cock Soda Lake sediments [35, 45], we have identified 1 archaeal and 219 bacterial MAGs that represent separate species based on average nucleotide identity (ANI) and are derived from prokaryotes with the potential for the dissimilation of sulfur compounds based on the presence of sulfur cycle marker genes (Fig. 4; Additional file 7: Dataset 3). Some MAGs were assigned to unexpected taxonomic and functional groups (summarized in Table 1).

**Table 1:** Description of high-quality MAGs from species representatives with novel metabolic potential

Name	GC	Cmpl	Cont	Novelty
*Rhodobacteraceae bac.* CSBr16_160	60	88	0.5	New genus of a photoheterotrophic SOB in the family *Rhodobacteraceae*. Genes for aerobic CO oxidation
*Gemmatimonadetes bac.* CSSed162cmB_429	68	99	3.9	First phototroph with the potential for carbon fixation in the phylum *Gemmatimonadetes* Putative role in the sulfur cycle (*soxCD*) and potential for dissimilatory nitrate reduction to ammonia
*Thiohalomonadaceae bac.* CSSed162cmB_532	61	92	1.7	New genus of colorless SOB in the family *Thiohalomonadaceae* (GTDB taxonomy). Genes for N_2_ fixation
*Flavobacteriales bac.* CSSed162cmB_293	53	95	0.5	New haloalkaliphilic genus within the *Bacteroidetes* with potential for S_2_O_3_^2−^ oxidation to S_4_O_6_^2−^ (*doxAD*)
“Ca. Woesearchaeota” *arch.* CSSed165cm_557	41	81	0	New genus within the *candidate* phylum Woesearchaeota. Putative S^0^/SO_3_^2−^ reducer
*Alkalilimnicola sp.* CSSed162cmA_191	67	87	0.9	Well-characterized haloalkaliphilic genus of SOB within the *Gammaproteobacteria*, new is potential for S_4_O_6_^2−^ reduction. Facultative autotroph and facultative anaerobe. Genes for aerobic CO oxidation and dissimilatory nitrate reduction to nitrite
*Geopsychrobacteraceae* bac. CSSed162cmA_454		94	1.9	New haloalkaliphilic genus within the class *Deltaproteobacteria*. Facultative anaerobe with potential for S_4_O_6_^2−^ reduction and N_2_ fixation

*GC* average G+C mol%, *Cmpl* % CheckM-completeness, *Cont* % CheckM-contamination

**Fig. 5:**
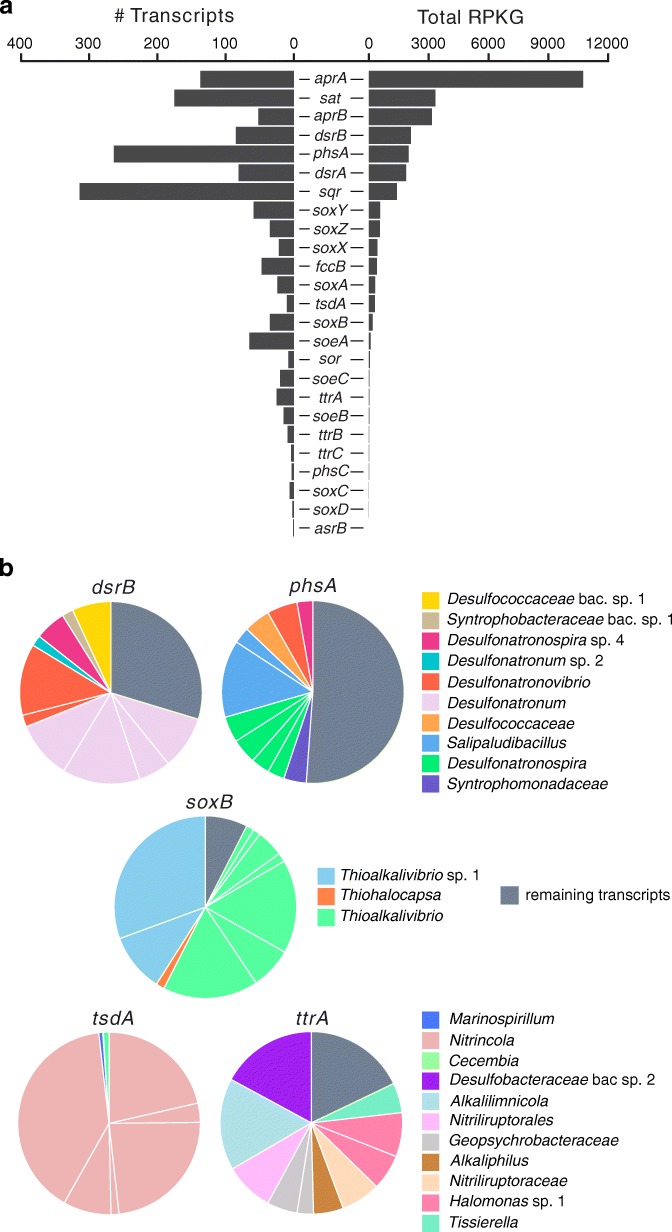
Abundance and taxonomic assignments of transcripts originating from sulfur cycle marker genes. **a** The number of unique transcripts (> 100 AA) and total abundance, expressed as reads per kilobase of sequence per gigabase of mapped reads (RPKG). RPKG was calculated for the complete contig on which each transcript was found and summed for each gene transcribed. **b** Taxonomic assignment of the top 10 most abundant transcripts of *dsrB*, *phsA*, *soxB*, *tsdA*, and *ttrA*. When a transcript was found to be 100% identical to a gene found on a MAG, the species assignment is given as a number

We obtained 1 metatranscriptome from the top 2-cm sediment layer [46], while from the deeper sediment layers, only a small amount of RNA could be extracted. Although ribosomal RNA (rRNA) was removed from the metatranscriptome before sequencing, still about ~ 42% reads were of ribosomal origin. In addition, several other structural, non-coding RNAs (ncRNA) were sequenced, most abundantly bacterial ribonuclease P (RnaseP) class A (~ 7% of ncRNA), transfer-messenger RNA (tmRNA, ~ 3%), and Ornate Large Extremophilic RNA (OLE RNA, ~ 1%; Additional file 8: Figure S4). Metatranscriptomic reads not originating from rRNA were assembled into 1,056,676 contigs of minimum 200 bp length, comprising in total 1,419,467 coding sequences [47]. Within KEGG, 17,880 hits were found to enzymes involved in energy metabolism, among which 1334 involved in nitrogen metabolism and 1717 in sulfur metabolism (Additional file 9: Figure S5). A total of 1498 unique transcripts (contigs > 100 AA) gave hits to an extended set of marker genes for dissimilatory sulfur cycling (Fig. 5).

### SRB and thiosulfate/polysulfide reductases

All species representatives that encoded the full canonical pathway for sulfate reduction (*sat*+*aprAB*+*dsrAB*) were *Deltaproteobacteria* (Fig. 4). Genes for a quinone-interacting membrane-bound oxidoreductase complex (*qmoABC*), which is suggested to be essential for sulfate reduction in deltaproteobacterial SRB [48], were present in these MAGs as well (Additional file 10: Dataset 4). *AprA*, *aprB*, *sat*, and *dsrB* ranked as the most abundantly transcribed marker genes for dissimilatory sulfur transformations that were investigated (summed RPKG of individual metatranscriptomic contigs; Fig. 5a). The majority of the *dsrB* transcripts originated from *Deltaproteobacteria* and especially known haloalkaliphilic lithotrophic SRB (Fig. 5b). We found a highly active transcription of *dsrB* assigned to a putative new genus within the family *Desulfococcaceae* (GTDB taxonomy, former *Desulfobacteraceae*) and transcribed from the corresponding MAGs that seem most closely related to the genus *Desulfonema* based on the phylogeny of 16 ribosomal proteins (Additional file 6: Dataset 2). Although less abundant, transcripts from a reversed type DSR assigned to gammaproteobacterial SOB were also recovered. The relative abundance of 16S rRNA gene transcripts assigned to several deltaproteobacterial SRB groups (*Desulfobacteraceae*, *Desulfonatronovibrio*, *Desulfonatronospira*, *Desulfonatronobacter*) was higher in the 0–2-cm compared to the 2–4-cm layer of sediments (Additional file 5: Figure S3).

Most of the deltaproteobacterial MAGs encoded for dissimilatory ammonifying nitrite reductases (*NrfAH*; Additional file 10: Dataset 4). A partial dissimilatory periplasmic nitrate reductase (*napA*) was only encoded by MAGs assigned to *Desulfonatronum*, *Desulfurivibrio*, and *Desulfobulbaceae* (GTDB taxonomy). The MAGs assigned to *Desulfonatronum*, *Desulfonatronovibrio*, and *Desulfonatronospira*, which are the genera that include haloalkaliphilic lithoautotrophic SRB also capable of thiosulfate/sulfite disproportionation [7], encoded for a thiosulfate/polysulfate reductase chain A (*phsA*). Our MAGs affiliating with *Desulfurivibrio*, a genus known for thiosulfate/polysulfide reduction and disproportionation, lacked the *phsA* gene. KEGG orthologs for the *phs* B and C subunits were not found in general. The *phsA* gene (but no *sat/apr* genes) was present also in putative species within the *Alpha*- and *Gammaproteobacteria* and several taxonomic groups not usually associated with the sulfur cycle, such as the phyla *Verrucomicrobia* (*Opitutae*), *Deinococcus-Thermus* (*Trueperaceae*), *Bacteroidetes* (*Bacteroidales*), *Actinobacteria* (*Egibacter*, *Nitriliruptoraceae*, *Nitriliruptorales*), *Ignavibacteria* (*Ignavibacteriales*), and *Spirochaetes* (*Spirochaetaceae*, *Spirochaetales*). In line with the metagenomic evidence, the transcripts from *phsA* originated from a wide diversity of organisms, but the most abundant transcripts were assigned to the members of the *Firmicutes* (most closely affiliated with *Salipaludibacillus* and *Syntrophomonadaceae*) or the *Deltaproteobacteria*.

One putative archaeal species within the phylum “*Candidatus* Woesearchaeota” (DPANN superphylum) encoded for the catalytic B subunit of the dissimilatory sulfite reductase found previously in sulfite-respiring enteric bacteria [49] (*asrB*; Table 1). The location of *asrB* in the genome suggests an important role within the central metabolism of this organism, as it was encoded alongside an acetate kinase (*ackA*) and phosphate-acetyltransferase (*pta*), a pyruvate dehydrogenase, a NADH:ubiquinone oxidoreductase, a putative sulfhydrogenase subunit delta, a Ni/Fe hydrogenase subunit alpha, and a hydrogenase formation chaperone (HypC/HypG/HupF family). “Ca. Woesearchaeota” was identified among the top 50 most abundant genera in the brine and sediments of Cock Soda Lake (Fig. 2), but no *asrB* transcripts were found in the metatranscriptome.

### Colorless SOB and purple sulfur bacteria strongly link cycling of sulfur to carbon and nitrogen fixation

Besides *Thioalkalivibrio*, several potential novel autotrophic SOB species were detected within the *Gammaproteobacteria* that encoded for the incomplete Sox-enzyme system without SoxCD (Fig. 4). In combination with a sulfide:quinone reductase (*sqr*) or a flavocytochrome c sulfide dehydrogenase (*fccAB*), a reversed dissimilatory sulfite reductase (*dsrAB*) and a sulfite-quinone oxidoreductase (*soeABC*) can be used for the complete oxidation of HS^−^ or other reduced sulfur compounds with formation of a zero-valent sulfur intermediate coupled to carbon fixation (*prkB*, *rbcLM*). All MAGs encoded at least for a cbb_*3*_-type cytochrome *c* oxidase, an additional aa_3_-type was encoded only by the species representatives from *Halorhodospira*, one unclassified *Gammaproteobacterium* and one member of *Thioalkalivibrio*. In none of the MAGs, the *fccA* gene (K17230) was found by GhostKOALA, but in most instances when a *fccB* gene was present (K17229), an adjacent gene encoding for cytochrome *c* subunit was identified by BlastP.

All sequenced *soxB* transcripts were assigned to the members of the *Proteobacteria.* The most abundantly transcribed genes were assigned to the chemolithoautotrophic genus *Thioalkalivibrio*, secondly to purple sulfur bacteria of the genus *Thiohalocapsa* (Fig. 5b)*.* The capacity for *NO*_*x*_ reduction within the genus *Thioalkalivibrio* differed between the putative species, some encoded near-complete pathways for denitrification, and others encoded dissimilatory nitrate reductases (*nap* or *nar*) and others a partial ammonifying nitrite reductase (*nrfA*; Additional file 10: Dataset 4). The species representative MAG of *Thiohalocapsa* encoded for a full ammonifying nitrite reductase (*nrfAH*), but again no *nar* or *nap* genes were present.

One species within the *Thiohalomonadaceae* (GTDB taxonomy) could not be assigned to a known genus and might affiliate with a novel, uncharacterized genus of chemolithoautotrophic SOB related to the genus thiodenitrifying *Thiohalomonas* [50] (previously within the family *Ectothiorhodospiraceae*; Table 1). Although no genes for denitrification were found, the capacity for nitrogen fixation (*nif* genes together with regulatory genes: *rnf* genes, *dra* genes, and a toxin-antitoxin system; Additional file 10: Dataset 4) was encoded, which is unusual for a colorless SOB. The reconstructed MAGs from the purple sulfur bacteria (encoding for *puf* and *bch* genes) genera *Thiohalocapsa* and *Halorhodospira* also encoded the capacity for nitrogen fixation, which is a typical trait for this functional group [42]. The third group of detected purple sulfur bacteria consisted of two species within the family *Chromatiaceae* not affiliated with known genera. None of these *Chromatiaceae* MAGs contained the *soe* genes, but sulfite oxidation could occur via an adenylylsulfate reductase (*aprAB*) and sulfate adenylyltransferase (*sat*). It should be noted that the other alternative enzyme for sulfite oxidation (sulfur oxygenase reductase (*sor*)) was only found in MAGs from the genus *Thioalkalivibrio*.

In addition, several more putative species within the *Gamma*- and *Alphaproteobacteria* encoded for a part of the Sox-system (*soxB*, *soxXA and/or soxYZ*), in combination with either *soeABC* (*Rubritepida*, *Geminicoccales*, *Alkalilimnicola*), *dsrB* (*Saccharospirillaceae*), both *soeABC* and *dsrAB* (*Thiorhodovibrio*, *Sedimenticolaceae*, and *Gammaproteobacteria*), or without the presence of other marker genes for dissimilatory sulfur oxidation (*Methylophaga*, *Methylomicrobium*, *Thiothrichales*, *Rhizobiales*, *Geminicoccaceae*, *Aquisalimonadaceae*, and *Pseudomonadales*). This can indicate either a partial acquisition or a partial loss of the whole sulfur-oxidizing gene repertoire, or be the result of existing gaps in the reconstruction of the MAGs. Some of the MAGs assigned to *Pseudomonadales* and *Sedimenticolaceae* also encoded near-complete pathways for denitrification (Additional file 10: Dataset 4).

### Photoheterotrophic SOB encoding *soxCD*

A full set of Sox-enzymes (including SoxCD), indicative of the potential for full thiosulfate oxidation to SO_4_^2−^ without the formation of intermediary zero-valent sulfur, was encoded by the members of the genera *Natronohydrobacter* and *Rhodobaca* and by one species representative of an unknown genus within the family *Rhodobacteraceae* (class *Alphaproteobacteria*; Table 1)*.* One MAG from the *Rhodobacteraceae* family encoded for a polysulfide/thiosulfate reductase (*psrA*/*phsA* gene). All other MAGs including from the genera *Roseinatronobacter* and *Roseovarius* encoded just a partial set of Sox-enzyme, probably because the MAGs were not complete. The absence of RuBisCo and the presence of genes for anoxygenic photosynthesis (*puf* and *bch* genes; Additional file 10: Dataset 4) in these MAGs suggest a photoheterotrophic lifestyle, which is consistent with the genomes obtained from cultured isolates [51]. These organisms might obtain additional energy from the oxidation of carbon monoxide as most MAGs encoded for an aerobic CODH (coxL, coxM, coxS). All MAGs encoded for two types of cytochrome *c* oxidases, with the exception of the putative species within *Roseinatronobacter* that encoded only for an aa_3_-type cytochrome *c* oxidase.

One species that affiliated with the *Dehalococcoidia* (phylum *Chloroflexi*) and four species of *Gemmatimonadetes* had *soxCD* genes but did not encode for other Sox-enzymes, and it is not clear what the role of the SoxCD might be in this case. At least in the well-established cases of the Sox system functioning in the proteobacterial SOB, SoxCD is never found alone. Three *Gemmatimonadetes* species were likely phototrophic (*puf* and *bch* genes) and encoded the capacity for dissimilatory nitrate reduction (*napAB* and *nrfAH*) to ammonia (Additional file 10: Dataset 4). One putative species had the additional potential capability for autotrophic carbon fixation (phosphoribulokinase, *prkB*; RubisCo type I, *rbcLM*; Table 1; Additional file 11: Figure S3) encoded in the genome. The presence of a putative aa3-type cytochrome c oxidase (*coxABCD*) further suggests an aerobic type of metabolism for this organism (Additional file 10: Dataset 4). Several MAGs from putative photoheterotrophic SOB (*Rhodobaca*, *Rhodobacteraceae*, *Roseinatronobacter*) and those assigned to the *Dehalococcoidia* encoded the capability for dissimilatory nitrate reduction to nitrite (*narGH*), but not for dissimilatory nitrite reduction to ammonia (DNRA) or further denitrification (Additional file 10: Dataset 4).

### Thiosulfate dehydrogenase-encoding heterotrophs

Four groups within the *Gammaproteobacteria* (*Thioalkalivibrio*, *Pseudomonas*, *Marinospirillum*, *Halomonas*), one within the *Bacteroidetes* (*Cecembia*), and one within the *Chlamydiae* (*Waddliaceae*) encoded for a thiosulfate dehydrogenase (*tsdA*; Fig. 4). Only in the gammaproteobacterial MAGs we identified adjacent to *tsdA* a gene for a cytochrome *c*4 (BlastP) that can function as an immediate electron acceptor for the two-electron oxidation of S_2_O_3_^2−^ to S_4_O_6_^2^. For several gammaproteobacterial heterotrophs, including haloalkaliphilic *Halomonas* species, thiosulfate oxidation is suggested to be used as an additional energy source to organotrophic growth and the produced S_4_O_6_^2−^ can chemically oxidize HS^−^ [26]. All *tsdA*-encoding MAGs had the potential for aerobic respiration and encoded a cbb_3_-type terminal oxidase. The MAGs assigned to *Cecembia*, *Halomonas*, and *Pseudomonas* encoded for an additional aa_3_-type. One putative *Halomonas* species detected here also encoded for a near-complete denitrification pathway (*narGH*, *nirS*, *norBC*, *nosZ*; Additional file 10: Dataset 4). The most actively transcribed *tsdA* genes in the top 2-cm layer of sediments could not be traced back to any of the reconstructed MAGs, but affiliated with c-type cytochromes from *Nitrincola* sp. A-D6 (80–92% AA identity; Fig. 5b). The remaining two transcripts were from the members of the genera *Marinospirillum* (*Gammaproteobacteria*) and *Cecembia* (*Bacteroidetes*). *Halomonas* might be more abundant and active in deeper sediment layers, as OTUs assigned to this genus were the most abundant in the 16S rRNA gene amplicons recovered from below 10 cm of depth (relative abundance ~ 15%, 21%, and 23% in the 10–15-cm, 15–20-cm, and 20–25-cm layers of sediment, respectively; Fig. 2).

The potential for thiosulfate oxidation using a thiosulfate:quinone oxidoreductase (DoxAD) as done by some acidophilic SOB and sulfur-oxidizing archaea [23] was also found in three species of *Bacteroidetes* (*Lishizhenia*, *Cryomorphaceae*, and *Flavobacteriales*; Fig. 4, Table 1). Transcripts of the *doxAD* genes were not found (Fig. 5), although genes for DoxX family proteins, which besides DoxD include mostly uncharacterized proteins wrongly annotated by GhostKOALA as DoxD (K16937), were abundantly transcribed.

### Widespread and actively transcribed putative tetrathionate reductase genes

A variety of different phylogenetic and functional groups encoded for a complete or partial tetrathionate reductase (*ttrABC*; Fig. 4). Complete tetrathionate reductases were encoded by three putative species within the *Gammaproteobacteria* (*Alkalilimnicola*, Table 1; *Halomonas*; and *Ectothiorodospiraceae*), one species within the *Bacteroidetes*, six species within the *Actinobacteria* (*Nitriliruptoraceae* and *Nitriliruptorales*), and three species within the *Deltaproteobacteria* (*Desulfobacteraceae*/*Desulfococcaceae*, *Desulfosarcinaceae*, and *Geopsychrobacteraceae*, Table 1). In the MAGs assigned to the three putative gammaproteobacterial species and to the *Desulfococcaceae*, an anoxia-responsive global transcriptional regulator (*fnr*, CPR/FNR family) was encoded, which was found to be essential for transcription of the *ttr* genes in *Salmonella typhimurium* [52]. In the MAGs affiliating with *Alkalilimnicola* and *Ectothiorhodospiraceae*, a *ttrS* gene was found near the *ttrABC* genes, which is part of a two-component regulatory system for tetrathionate respiration. Although overall *ttrA* transcription was low compared to the other marker genes (Fig. 5a), transcripts were assigned to a variety of taxonomic groups within the *Deltaproteobacteria*, *Gammaproteobacteria*, and *Actinobacteria* also detected in the metagenomes, as well as to the *Firmicutes* (*Alkaliphilus* and *Tissierella*).

## Discussion

By high-throughput sequencing, we confirmed the presence and transcriptional activity of many cultured groups of prokaryotes and potential novel groups that dissimilate inorganic sulfur compounds in the moderately hypersaline (55 g L^−1^ total salt) Cock Soda Lake, confirming previous studies that a complete sulfur cycle between SO_4_^2−^ and HS^−^ can occur at such a salinity level in soda lakes [8]. Most of the nucleic acids were recovered in the largely anoxic top few centimeters of sediments, which strongly suggests that the sediment-brine interface and the partially oxidized sediment surface layer were a hotspot for microbial growth and activity. In this top 2-cm layer of the sediments, heterotrophs, lithotrophic SRB, purple sulfur bacteria, and chemolithoautotrophic SOB were transcriptionally active and had the potential to tightly link the biogeochemical cycles of carbon, nitrogen, and sulfur. Many members of the latter three functional groups had the ability to fix nitrogen in addition to inorganic carbon. The majority of the deltaproteobacterial SRB encoded the capacity for DNRA (*nrfAH*), although mostly decoupled from dissimilatory nitrate reduction to nitrite. The possibility for growth by sulfide oxidation coupled to dissimilatory nitrate reduction to ammonia by the members of *Desulfonatronum*, *Desulfurivibrio*, and a novel genus within the *Desulfobulbaceae*, for which we recovered MAGs encoding additionally for *napA*, warrants further investigation, as this process was recently shown for *Desulfurivibrio alkaliphilus* [16]. Also in some SOB MAGs assigned to *Thioalkalivibrio* and a MAG recovered from *Thiohalocapsa*, dissimilatory nitrite reductases were found (nirKS and nrfAH, respectively), but not together with nitrate reductases in the same genome. Marker genes for complete denitrification were found mostly in gammaproteobacterial MAGs, including putative chemolithoautotrophic and lithoheterotrophic SOB. Putative heterotrophic and denitrifying SOB from the genus *Halomonas* seem to be relatively more important deeper in the sediments. There, the type and amount of organic substrates as well as the extreme low redox potential are perhaps less favorable for other heterotrophs, such as the members of the ML635J-40 aquatic group (*Bacteroidetes*) that are dominant in the top layer of sediments and presumably contribute to the degradation of dead phototrophic biomass [53].

High amounts of methane were detected in the brine that exceeded the concentrations measured in the sediment. It is not clear if this methane was produced by methanogens in the anoxic sediment layers and later trapped in the brine, or if aerobic methane production in the brine itself occurred. We also obtained one MAG of a putative methanotroph from the 0–5-cm sediment layer assigned to the genus *Methylomicrobium*, from which halo (alkali) philic methanotrophs have been previously isolated [54]. On the other hand, we could not detect free HS^−^ in the brine or down to 25-cm sediment depth. This suggests HS^−^ is rapidly turned over by SOB in the top layers, chemically oxidized, or bound as FeS in the deeper layers. We showed that the intermediate S_2_O_3_^2−^ is rapidly oxidized by SOB from the sediment top layer under oxic conditions, but found that when the top 2-cm sediment was amended with millimolar concentrations of S_2_O_3_^2−^, oxidation was partially inhibited under light conditions. Since this phenomenon did not occur when the sediments were amended with micromolar concentrations of S_2_O_3_^2−^, we infer that the light-inhibited SOB are those using enzymes with low S_2_O_3_^2−^ affinity. The lower rates of thiosulfate conversion obtained for the 2–4-cm sediment layer is presumably the result of an overall decline in microbial biomass with sediment depth.

Also, the reductive part of the sulfur cycle is likely primed to the top 2 cm of sediments where most of the DNA and RNA were recovered. A previous study found a steep drop in the sulfate reduction rates below 2 cm of sediment depth determined in situ for Cock Soda Lake [31], which is also in agreement with our sulfate concentration profiles and the relatively higher 16S rRNA gene transcripts assigned to SRB found in the 0–2-cm layer compared to the 2–4-cm layer. Further, we found the widespread presence and transcription of a subunit of putative thiosulfate/polysulfide reductase homologs in the top layer of sediments, including with affiliation to some taxonomic groups not previously associated with the sulfur cycle. This did not coincide with the depletion of S_2_O_3_^2−^ under anoxic conditions, as we were able to measure micromolar concentrations of S_2_O_3_^2−^ in the pore brine of the top 4-cm sediment layer. This could indicate that the substrates for these reductases are mostly S_*n*_^2−^, which are stable compounds under anoxic, haloalkaline conditions and were previously shown to be the preferred substrate at least for isolated SRB [55]. On the other hand, we cannot exclude that the detection of the partially oxidized sulfur intermediates S_2_O_3_^2−^ and SO_3_^−^ might have been an artifact from the pore water extraction procedure. A somewhat surprising finding was that despite the chemical instability and reactivity of S_4_O_6_^2−^ with HS^−^ at high pH [26], full tetrathionate reductases were transcribed by several anaerobic and facultative anaerobic *Bacteria.* This could mean that the sulfur intermediate S_4_O_6_^2−^ is biologically available as an electron acceptor at an alkaline pH, perhaps in this case because HS^−^ is rapidly consumed by other processes.

Compared to the MAGs reconstructed previously from sediments of Kulunda Steppe soda lakes [35], a total of 12 MAGs were recovered in this study from 3 extra phyla, namely the *Oligoflexia* (*Bdellovibrionota*), “*Candidatus* Delongbacteria,” and from the “*Candidatus* Lokiarchaeota” within the superphylum *Asgardarchaeota*, an archaeal group from which the eukaryotes are envisioned to have emerged [56]. Some of the most transcriptionally active organisms in the dissimilatory sulfur cycle were not previously recognized to be haloalkaliphiles [7], such as purple sulfur bacteria from the genus *Thiohalocapsa* and a new species of SRB from the family (*Desulfococcaceae*/*Desulfobacteraceae*). Additionally, several new MAGs were obtained with some unusual metabolic potential for specific taxonomic groups. First, we found a MAG from a member of the phylum *Gemmatimonadetes* with the capacity for photoautotrophy, while the single characterized phototroph from this phylum is a photoheterotroph [57, 58]. Second, we found the capacity for nitrogen fixation in a colorless SOB from the family *Thiohalomonadaceae*, which is a remarkable trait for this functional group. To our knowledge, only few free-living colorless SOB from the genus *Thiothrix* [59] and 1 marine, chemoautrophic mollusk symbiont [60] are known to perform nitrogen fixation. Third, we found a MAG from the “Ca. Woesearchaeota” with an encoded subunit of a sulfite reductase (*asrB*), which is peculiar as the phylogenetic distribution of this enzyme is thought to be restricted to the domain *Bacteria* [61]. The genomic setting of *asrB* exhibited strong similarity with that in the genome of the sulfur-reducing hyperthermophilic, heterotrophic archaeon *Pyrococcus furiosus* [62] and should deserve further attention. Previously, an anaerobic heterotrophic lifestyle and a symbiotic association with methanogens have been suggested for most members of the “Ca. Woesearchaeota” [63]. Although this study included a wide range of oxic and anoxic environments globally, it did not yet include the most recent 23 MAGs of haloalkaliphiles ([40, 45]) that seem to occur abundantly both in soda lake brines and sediments. We identified here at least 1 species of the “*Candidatus* Woesearchaeota” that might be capable to reduce sulfur compounds.

In general, more detailed genome analysis and physiological assays are needed to better understand the genetic context and the function of hypothetical proteins, but these are beyond the scope of this paper. In addition, metagenomes are a snapshot in space and time, and metatranscriptomes even more so. Since we were only able to obtain one metatranscriptome from the sediment surface layer, many open questions remain. Not only would it be preferable to compare the transcription under light versus dark conditions, our failure to obtain sufficient nucleic acids from below 5-cm sediment depth is an invitation to investigate the sediment top layer at a finer scale in future studies and to collect far larger volumes of sediment from the deeper layers for nucleic acid extraction.

## Conclusion

We uncovered the presence of new haloalkaliphilic prokaryotes that still evade culturing efforts, some with unusual metabolic potential. A new genus of colorless SOB from the family *Thiohalomonadaceae* might be capable of fixing nitrogen, which is a rare trait for this functional group. We obtained MAGs from a new species within the “Ca. Woesearchaeota” with the potential for elemental sulfur/sulfite reduction. Although its role in the sulfur cycle is less certain, we found one new species within the recently characterized phylum *Gemmatimonadetes* with genes indicating the capacity for autotrophy and aerobic anoxygenic phototrophic growth. We found that the sediment microbial community oxidized S_2_O_3_^2−^ rapidly in the presence of oxygen, but that oxidation was partially inhibited by light. Although putative thiosulfate/polysulfide reductases were highly transcribed by a great variety of taxonomic groups, including some not usually associated with inorganic sulfur compound cycling, we could still measure S_2_O_3_^2−^ in the sediment pore water. Finally, despite its reactivity, we found that S_4_O_6_^2−^, an intermediate of sulfur oxidation unstable at soda lake conditions, could potentially also be used as an electron acceptor by many functional groups of the soda lake prokaryotic community.

## Methods

### Sampling, preservation and in situ measurements

During a fieldwork survey in July 2016, samples were taken in the littoral zone of Cock Soda Lake (Kulunda Steppe, southwestern Siberia, Altai, Russia). On site, the total salinity in the brine was determined with a refractometer. Brine alkalinity was determined by titrating first sodium carbonate to bicarbonate in the presence of phenolphthaline (pH ~ 8), then the total bicarbonate (i.e., originating from carbonate + the original pool) to carbonic acid with 1 M HCl in the presence of methyl orange (pH ~ 4.5). One 20-cm-long sediment core was processed in the field into 5-cm-thick slices for the determination in the laboratory of acid-volatile sulfide content (FeS), free sulfides, and methane in the pore water as described by [64]. Additional small samples (~ 2 mL) from each layer of this column were well mixed, later flash-frozen in the laboratory, and stored at − 20 °C for DNA isolation. In order to target primarily the planktonic prokaryote communities, brine samples were filtered through 5-μm pore-sized syringe filters to remove phytoplankton cells and large particles. The filtrate was mixed with an equal portion of sterile DMSO (20% *v*/*v*)-EDTA (0.25 M) salt-saturated buffer (DESS [65]) to preserve the microbial communities upon transport to the laboratory. Three sediment cores of 12 cm length (Ø 6 cm) were cut into 2-cm-thick layers and well mixed. Smaller subsamples (3 mL ~ 6 g wet weight) from each layer were stored with 12 mL “LifeGuard Soil Preservation Solution” (MO BIO) directly in bead beating tubes to preserve the DNA and RNA upon transport to the laboratory. The remaining sediments from each layer were combined, flash-frozen with liquid nitrogen after transport to the laboratory, and stored at − 80 °C both for DNA isolation and determination of general sediment characteristics.

### General sediment characteristics

Sediment samples were freeze-dried, and dry weight was determined using a four-decimal scale. After treatment with hydrogen peroxide and hydrochloric acid, standard grain size analysis was applied using a set of mesh sieves for the fraction > 125 μm and a sedigraph (Sedigraph III Plus, Micrometrics) for the fraction < 125 μm. Total carbon content was determined by dry combustion with an elemental analyzer (Elementar Vario El Cube, Germany). Inorganic carbon was determined based on gravimetric loss of carbonates as carbon dioxide in the presence of excess hydrochloric acid [66]. After microwave-assisted (PAAR Physica multiwave) digestion in *Aqua regia*, total sulfur, phosphorus, manganese, and iron concentrations in the sediment were measured using an ICP-OES (Perkin Elmer Optima 8000 crossflow).

### Biogeochemical profiling

Three intact replicate cores were transported back to the laboratory for chemical analyses of the pore water, potential activity measurements, and microsensor measurements. Before measuring, the columns were kept for 1 week at constant temperature (20 °C) under a 16:8-h day/night light regime to allow displaced pore water ions to equilibrate. Detailed oxygen and redox (oxidation-reduction potential against a silver/silver-chloride reference electrode) profiles of the top layer sediment were measured at three different spots of each core, manually maneuvering microsensors with a 25-μm glass tip (Unisense, Aarhus, Denmark) from the top of the sediment downwards. pH profiles were measured once for two columns. The electrodes were connected to a “Microsensor Multimeter” (Software Unisense Microsensor), calibrated in the appropriate pH range and handled according to the manufacturer’s instructions.

After the microsensor measurements, the pore water from one sediment column was extracted from 30-cm^3^ subsamples of the top 0–2-cm and 2–4-cm layers by centrifugation (1 h at 19000×*g* and 4 °C). After 0.2-μm filtration, half of the pore water was fixed with zinc acetate (5% *w*/*v* final concentration, to trap free sulfides that might damage the column) for thiosulfate, sulfate, nitrate ,and chloride determination with an ion chromatograph (IC). The other half was used for thiosulfate and sulfite determination. The samples were stored at − 80 °C until analysis.

A second sediment column was further used for activity measurements. The top 0–2-cm and 2–4-cm layers of the sediment were mixed well, and 1-mL subsamples were added to sterile 50-mL flasks with a sterile cutoff syringe. To measure thiosulfate (S_2_O_3_^2−^) consumption under oxic conditions, 14 mL of 0.2-μm-filtered natural brine was added and bottles were closed with cotton stoppers. The live sediment slurries were placed on a shaker and allowed to acclimatize overnight either under white light (15 W “Edmund Bühler TH30 Incubator Hood,” measured PAR photon flux between 18 and 33 μmol m^−2^ s^−2^ with “Li-Cor Light Meter” model LI-250) or in the dark. Dead controls were established by autoclaving the sediments three times at 120 °C for 20 min. To determine the consumption in the micromolar range, duplicate bottles for each sediment layer and pre-treatment were spiked with final concentrations of 200 μM S_2_O_3_^2−^. All bottles were sampled immediately after spiking (*t*_0_) and after 8, 24, and 48 h of incubation. To determine thiosulfate consumption in the millimolar range, the thiosulfate-exposed slurries were spiked a second time to a final concentration of 3 mM S_2_O_3_^2−^ after replenishing the liquid phase with sterile brine to 19 mL and overnight acclimatization. The incubations were sampled immediately after the second spike (*t*_0_) and after 3, 6, 12, 24, and 48 h of incubation. Before sampling, all bottles were stirred thoroughly by hand after which large particles could settle briefly. All samples were taken from the liquid phase and filtered through a 0.22-μm PES membrane. Samples for the UPLC (micromolar range) were flash-frozen with liquid nitrogen and stored at − 80 °C. Triplicate samples for the IC (millimolar range) were fixed in excess zinc acetate (1.5% *w*/*v* final concentration) and stored at − 80 °C.

### Sulfur compound and methane analyses

Acid-volatile sulfide (FeS) and free sulfides were determined with the methylene blue method according to [55]. Methane concentrations were measured using gas chromatography according to [64]. Thiosulfate and sulfite concentrations were determined by ultra-performance liquid chromatography (UPLC) after monobromobimane (MBB) derivatization using a method from [67], but modified for alkaline conditions. Thiosulfate and sulfite standards were prepared in the sulfate-free, degassed artificial soda medium. Samples were thawed, sonicated for 10 min in an ultrasound bath, and transferred to dark glass vials. To lower the pH to 8 and allow adequate reaction of thiosulfate with MBB, 50 μL of concentrated methanesulfonic acid (MSA, 1.5 M) was added to 200-μL samples and standards. After letting the preparations briefly degas, samples and standards were derivatized in the dark with a freshly prepared derivation mixture (25 μL 10 mg/mL MBB in acetonitrile and 25 μL 500 mM HEPES-50 mM EDTA buffer pH 8) for 30 min at room temperature. The reaction was stopped by adding 50 μL concentrated MSA. Derivatized samples and standards were analyzed on a “Waters Aquity” UPLC (Aquity UPLC BEH C8 1.7 μm, 2.1 × 50 mm column) with fluorescence detection (excitation 380 nm, emission 480 nm). Sulfate and thiosulfate concentrations in diluted samples (total 60×) were determined on a “Metrohm” ion chromatograph using electronically background conductivity suppression and conductivity detection. Fresh standards were prepared daily.

### Nucleic acid extraction and processing

Simultaneous extraction of DNA and RNA from “Lifeguard”-preserved samples (triplicates) was performed with the “RNA PowerSoil® Total RNA Isolation Kit” (MO BIO). The DNA in the total RNA was digested with DNase I (“Turbo DNA-free kit,” Ambion), and no further residual 16S rRNA gene contamination could be detected after a 30-cycle PCR (Additional file 12: Figure S7). For amplicon sequencing of the 16S rRNA gene transcripts, DNA-free RNA was transcribed into single-stranded cDNA using random hexamer primers (“SuperScript First Strand synthesis kit,” Invitrogen). Additional DNA was isolated from flash-frozen sediment samples and the DESS-preserved brine sample using the “PowerSoil DNA isolation kit” (MO BIO) and further cleaned with a “Genomic DNA Clean & Concentrator kit” (Zymo). The brine/DESS mixture was first filtered onto 0.2-μm pore-sized sterile membrane filters. Sediment samples and filter pieces were first incubated at 65 °C under light shaking in Solution S1 before proceeding to further DNA extraction according to the manufacturer’s instructions. All nucleic acids were checked on agarose gels and quantified with “Qubit” standard assays (Invitrogen).

### 16S rRNA gene and transcript amplicon sequencing and analysis

Before amplicon sequencing, DNA and cDNA solutions were standardized to concentrations to 1, 5, or 20 ng/μL. At the sequencing center (MR DNA), 16S rRNA gene and transcript fragments were amplified using the 515F-Y/926R primer set (targeting the V3–V4 region) as described before [68]. The individual samples were barcoded, and the mixtures were sequenced on a “Illumina MiSeq” platform. The 2 × 300 bp paired-end amplicon sequence reads were merged and re-orientated, and the reads shorter than 200 b, primers, and adapters were removed. Reads ≥ 150 b were further quality trimmed using Sickle (default parameters; [69]), and the dataset was further processed with the automated NGS analysis pipeline of the SILVA rRNA gene database project (SILVAngs 1.3, db v132) [70]. The resulting OTUs (clustered at 98% identity) and assigned taxonomy (≥ 93% identity BLAST) were further analyzed with the package ampvis2 in “R” [71].

### Metagenomes and metatranscriptome sequencing

The DNA mixtures isolated from the brine (~ 3 μg), the top 0–2-cm sediment layer (~ 4 and 6 μg), and the top 0–5-cm layer (~ 6 μg) were used for direct metagenomics sequencing. At the sequencing center (Beijing Genomics Institute), the quality and integrity of the DNA were again verified before preparing short-insert (350 bp) libraries. The DNA was fragmented (Covaris), end-repaired (End Repair Mix), and purified (“QIAquick PCR Purification kit,” Qiagen). Adapters were ligated at 3′ adenylated ends of the DNA fragments, and adapter-ligated DNA fragments were enriched by several rounds of PCR amplification.

Only from the 0–2-cm top layer enough RNA (~ 7 μg) was isolated for direct metatranscriptome sequencing. The DNA-free RNA was pooled from triplicate extractions after checking its integrity on an agarose gel (Additional file 12: Figure S7). At the sequencing center (BGI), the total RNA concentration, the RNA integrity number (RIN), the 23S to 16S ratios, and the fragment size were verified with a “Agilent 2100 Bioanalyzer” (Agilent RNA 6000 Nano Kit). The purity of the samples was tested using “NanoDrop™” before preparing the prokaryotic strand-specific libraries (Illumina Truseq). A second DNase I digestion was performed, and rRNA was depleted (Ribo-Zero™ Magnetic Kit). After fragmentation, the first-strand cDNA was generated using random primers, and second-strand cDNA was synthesized and end-repaired. Adapters were ligated at 3′ adenylated ends of the cDNA fragments.

The quantity and quality of the DNA and cDNA libraries were checked again on the “Agilent 2100 Bioanalyzer” and with RT-qPCR using “TaqMan Probes” before preparing flow cells. Libraries were paired-end sequenced (2 × 150 bp) on a “Illumina HiSeq4000” platform, and low-quality reads were removed (40% of bases with *Q* < 20 or 50% adapter).

### Metagenomic analysis

Metagenomic reads were quality and length (> 21 b) trimmed with Sickle [69] and Illumina adaptor/vectors and primers removed with BBDuk [72]. Trimmed reads were assembled into ≥ 1 kb contigs using MEGAHIT (version v1.0.3-6-gc3983f9 [73], --k-min 21, --k-max 121, --k-step 10). Additional metrics on the raw sequence data and assembled contigs are given in Additional file 3: Table S3. Open reading frames were predicted with Prodigal [74], tRNAs were predicted with tRNAscan-SE [75], and rRNAs were predicted with rna_hmm3. The assembled 16S rRNA gene sequences were annotated using the SILVA SSU database v132 (*e* value ≥ 1e−5 [70]). The predicted protein sequences were annotated against the KEGG database with GhostKOALA [76].

Differential coverage binning of ≥ 2.5-kb contigs was done with Metabat2 (version 2.12.1 [77]; default settings). To improve the binning process, coverage was not only calculated for the metagenomics read sets sequenced for this study [46], but also for previously obtained metagenomes from Kulunda Steppe soda lakes [34, 35, 78–86] by mapping all read sets on the assembled contigs with BBmap (version v36.x [87]). Post-binning analysis on the resulting 2062 bins was done with Anvi’o [88] as described before [35], only without manually removing virus-related genes from the bins. Overall, 1032 novel metagenome-assembled genomes (MAGS) were obtained that were of reasonable quality (≥ 50% CheckM-completeness and < 10% contamination) and/or were assigned to the candidate phyla radiation (CPR; Additional file 4: Dataset 1) [40]. Taxonomic assignment of the individual MAGs was based on GTDB taxonomy [89] which uses concatenated protein phylogeny. The environmental abundance of the MAGs was estimated by calculating RPKG (= mapped reads per kb MAG sequence per Gb mapped reads) after mapping (> 95% similarity of > 25 bases) 10 million randomly generated read subsamples from all available Cock Soda Lake metagenomes, including 2 metagenomes previously obtained from the top 10-cm sediment layer sampled in 2010 and 2011 [35, 84, 85].

Among the newly obtained MAGs [40] and 401 MAGs previously obtained from Cock Soda Lake [35, 45], around 400 MAGs encoded marker genes (K number assignments) for dissimilatory sulfur compound transformations. We further simplified the MAG dataset based on assigned taxonomy and average nucleotide identity (ANI; [90]), retaining 263 MAGs as the best species representative (highest CheckM-completeness) for each putative sulfur-cycling group. When the GTDB taxonomy of these species representatives could not be assigned down to the genus level, the relatedness of these species representatives was further assessed based on the phylogeny of 16 ribosomal proteins as described previously [35] (Additional file 6: Dataset 2). For all the species representatives, more detailed comparative sequence and phylogenetic analyses were performed with reference protein sequences from the eggNOG database [91] to confirm the KEGG annotation, including characterized reference proteins for the following S-cycling genes: *doxD* [23], t*sdA* [24], *ttrA* [52], *soxB* [19], and *dsrB* [92]. For *doxD* and soxB, initial trees were calculated to discriminate the bonafide *doxD* and soxB sequences from *doxX* family proteins and 5′-nucleotidases, respectively. For *phsA*, reference protein sequences were retrieved from the NCBI protein database. The final phylogenetic trees are available through figshare [93]. Additionally, marker genes for central metabolic pathways and key environmental element transformations were identified based on K number assignments. A phylogenetic tree including the AA sequence for the large subunit of RubisCo of one *Gemmatimonadetes* MAG was constructed according to [35].

### Metatranscriptome analysis

Metatranscriptomic reads were quality and length trimmed, and contaminants and vectors were removed with BBDuk [72]. The reads were mapped against the assembled ≥ 1 kb metagenomics contigs (originating from the same sample) with Bowtie2 [94] for strand-specific libraries (--nofw) using the “very sensitive” mode and allowing one mismatch in the seed alignment. Since this resulted in a low poor alignment rate, we opted for de novo assembly of transcripts. The rRNA (LSU, SSU, 5S rRNA, and 5.8S rRNA) encoded on both read mates were first removed with SortMeRNA [95]. Other non-coding RNA (ncRNA) of interest (including tRNA, mtRNA, CRISPRs, OLE RNA) on the trimmed metatranscriptomic reads were identified using Infernal against the Rfam database (version 14.0; [96]) but were not removed from the read sets. Assembly was performed with MEGAHIT (v1.1.2 [73], --k-min 21, --k-max 121, --k-step 10) retaining contigs ≥ 200 b. Additional metrics on the raw metatranscriptome sequence data and assembled transcripts are given in Additional file 3: Table S4. Open reading frames on the resulting cDNA transcripts were predicted with Prodigal; predicted proteins were annotated with GhostKOALA [76] and scanned for S-cycling marker genes based on K number assignments. The KEGG annotations were confirmed by including the transcripts in the single gene trees (see the previous paragraph). Transcripts > 100 amino acids that were contained already in longer transcripts were de-replicated with dedupe.sh [97] (100% identity, *k* = 11). Relative abundances of the de-replicated S-gene transcripts > 100 amino acids were estimated by calculating RPKG for the full contigs after mapping (> 95% similarity of > 25 bases) 25% of the metatranscriptomic forward reads used for assembly. Where possible, S-gene transcripts were linked to the obtained MAGs and inferred species using blastp (100% identity). Taxonomy for the remaining de-replicated transcripts was inferred by blasting against the NCBI nr database and comparing the results with the taxonomic assignment by GhostKOALA.

## Additional files

Additional file 1: **Figure S1.** a) Nitrate and chloride ion concentrations measured in the brine and pore water samples. b) Inorganic carbon content of dried sediment samples with depth. (PDF 83 kb)

Additional file 2: **Figure S2.** pH profile measured on two replicate sediment columns. (PDF 105 kb)

Additional file 3: **Table S1.** Grain size distribution of the top 12-cm sediment layer of Cock Soda Lake. **Table S2.** Element composition of dry sediment determined by inductively coupled plasma mass spectrometry (ICP-MS). **Table S3.** Metrics on the metagenomic raw sequence reads and assembled contigs obtained from Cock Soda Lake in 2016. **Table S4.** Metrics on the metatranscriptomic raw sequence reads and assembled transcripts obtained from the top 2-cm sediment layer from Cock Soda Lake in 2016. (PDF 120 kb)

Additional file 4: **Dataset 1**. Metrics and Genbank accession numbers on the obtained metagenome-assembled genomes (MAGs). The total size of the MAG (Mb), the number of contains (# Contigs), the contig N50 (kb), maximum contig length (kb), estimated mean coverage, average G+C mol%, total number of 5S, 16S and 23S rRNAs, total number of tRNAs and estimated percentages of CheckM-completeness (Compl), CheckM-contamination (Cont), and strain heterogeneity (Str het) are given. The final given name used for the Genbank submission (NCBI organism name) was derived from the Genome Taxonomy Database (GTDP) classification according to [89] and where possible verified with the 16S rRNA gene classification according to the SILVA database (see column “Proposed name (GTDB)”). For the latter, BLAST results are reported: taxonomy of the best hit, identity with this best hit (%), *e* value of the match, the length of the query (number of nucleotides), and the coverage with the hit (%). (XLSX 182 kb)

Additional file 5: **Figure S3.** Relative abundance of top 50 abundant genera in the top 4 -cm sediment layer of Cock Soda Lake identified by amplicon sequencing of 16S rRNA genes and transcripts. (PDF 112 kb)

Additional file 6: **Dataset 2.** Phylogenetic tree of all bacterial MAGs obtained in this study and in [35] based on 16 ribosomal proteins. References included in the tree were obtained from [98]. (TRE 269 kb)

Additional file 7: **Dataset 3.** Species representative MAGs that encode for S-cycling marker genes. Basis for Fig. 3. KEGG number assignment, CheckM-completeness, and CheckM-contamination are given. X, gene absent; V, gene present. (XLSX 53 kb)

Additional file 8: **Figure S4.** Rfam family assignment of metatranscriptomic reads originating from non-coding RNA (ncRNA). Only the percentage of reads (relative to the total of 136474149 reads assigned as ncRNA) for the top 10 abundant Rfam families is shown. OLE RNAs are widespread among thermophilic Firmicutes, including sulfidogens from the genera Desulfotomaculum, but their exact function remains unknown [99]. (PDF 88 kb)

Additional file 9: **Figure S5.** Functional classification of the KEGG-annotated transcripts. (PDF 89 kb)

Additional file 10: **Dataset 4.** Presence-absence of marker genes for central metabolic pathways and environmentally relevant element transformations in a selection of MAGs. MAGs were selected based on the presence of markers for dissimilatory sulfur cycling. KEGG number assignment, CheckM-completeness, and CheckM-contamination, as well as species delineation (based on average nucleotide identity, see the “Methods” section) are given. X, gene absent; V, gene present. (XLSX 261 kb)

Additional file 11: **Figure S6.** Maximum likelihood tree showing the phylogeny of the large subunit of RubisCo found in the MAG of the putative photoautotrophic *Gemmatimonadetes* bacterium. (PDF 154 kb)

Additional file 12: **FigureS7.** Agarose gels showing the integrity of the total RNA used for metatranscriptomics sequencing (top 0–2-cm sediment layer) and amplicon sequencing of 16S rRNA gene transcripts (top 0–2-cm and 2–4 -cm sediment layers). (PDF 522 kb)

## Data Availability

All raw sequence reads from the metagenomes, metatranscriptome, and 16S rRNA gene and transcript amplicon sequencing were deposited to the NCBI Sequence Read Archive (SRA) under the accession number SRP144042 [46]. The final 1032 MAGs obtained in this study have been deposited as whole-genome shotgun projects at DDBJ/ENA/GenBank, and accession numbers are listed in Additional file 4: Dataset 1 (BioProject ID PRJNA453733) [40]. All versions described in this paper are version XXXX01000000. The transcripts assembled from the metatranscriptome (DOI:10.21942/uva.7819565) [47] and supporting phylogenetic analyses are available through figshare (DOI:10.21942/uva.c.4594961) [93]. All other datasets generated or analyzed during this study, such as sequence annotations and protein alignments constructed for phylogenetic trees, are available from the corresponding author on reasonable request.
