# Supplementary material for: Metagenomes and metatranscriptomes shed new light on the microbial-mediated sulfur cycle in a Siberian soda lake
Source: BMC Biol. 2019 Aug 22;17:69. doi: 10.1186/s12915-019-0688-7 (PMC6704655; doi:10.1186/s12915-019-0688-7)
Supplement: Supplementary file 1 — Figure S1. a) Nitrate and chloride ion concentrations measured in the brine and pore water samples. b) Inorganic carbon content of dried sediment samples with depth. (PDF 83 kb) [file 12915_2019_688_MOESM1_ESM.pdf]

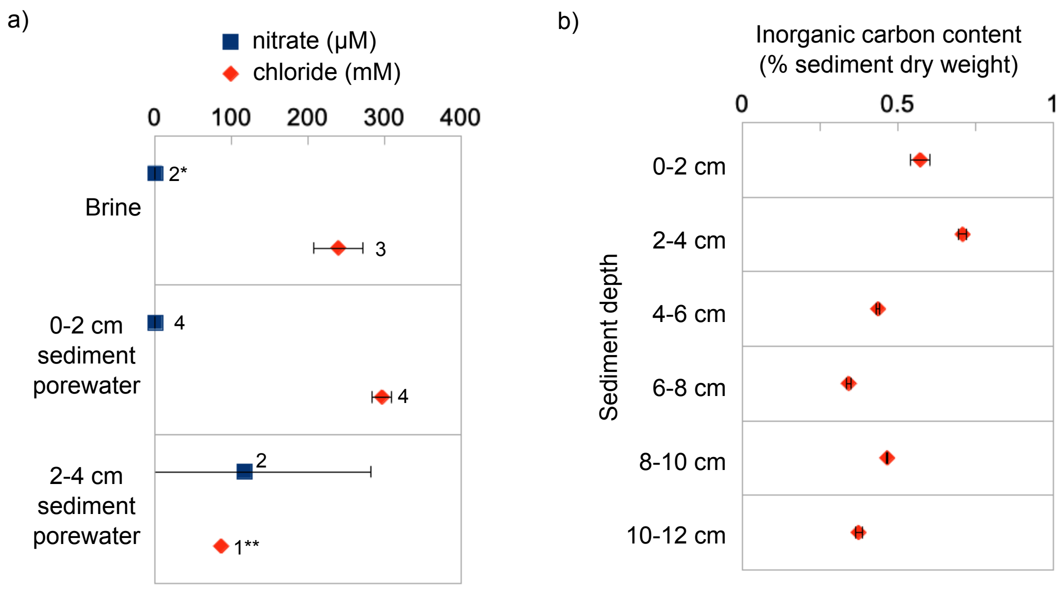

Supplementary Figure 1. a) Nitrate and chloride ion concentrations measured in the brine and porewater samples. Error bars show the standard deviation and the number of replicate measurements are given next to the data points. * In one out of three samples a clear nitrate peak was observed, but the area was outside of the calibration range. ** For one out of two samples, the chloride peak failed. **b) Inorganic carbon content of dried sediment samples with depth.** For each depth, two replicate measurements were performed. Error bars show the standard deviation.
