# Supplementary figures and images for: Metagenomes and metatranscriptomes shed new light on the microbial-mediated sulfur cycle in a Siberian soda lake
Source: BMC Biol. 2019 Aug 22;17:69. doi: 10.1186/s12915-019-0688-7 (PMC6704655; doi:10.1186/s12915-019-0688-7)

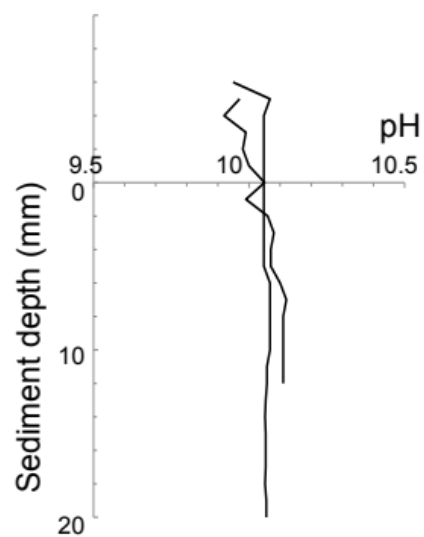

Figure S2 pH profile measured on two replicate sediment columns.

Supplement: Supplementary file 2 — Figure S2. pH profile measured on two replicate sediment columns. (PDF 105 kb) [file 12915_2019_688_MOESM2_ESM.pdf]
