# Supplementary material for: Metagenomes and metatranscriptomes shed new light on the microbial-mediated sulfur cycle in a Siberian soda lake
Source: BMC Biol. 2019 Aug 22;17:69. doi: 10.1186/s12915-019-0688-7 (PMC6704655; doi:10.1186/s12915-019-0688-7)
Supplement: Supplementary file 3 — Table S1. Grain size distribution of the top 12-cm sediment layer of Cock Soda Lake. Table S2. Element composition of dry sediment determined by inductively coupled plasma mass spectrometry (ICP-MS). Table S3. Metrics on the metagenomic raw sequence reads and assembled contigs obtained from Cock Soda Lake in 2016. Table S4. Metrics on the metatranscriptomic raw sequence reads and assembled transcripts obtained from the top 2-cm sediment layer from Cock Soda Lake in 2016. (PDF 120 kb) [file 12915_2019_688_MOESM3_ESM.pdf]

Table S1. Grain size distribution of the top 12 cm sediment layer of Cock Soda Lake.

Classification	Proportion sample (%)
Gravel	0.0
Sand	79.7
Mud	20.3
Coarse sand	2.2
Medium sand	29.9
Fine sand	9.4
Very fine sand	38.2
Very coarse silt	10.6
Coarse silt	3.1
Medium silt	2.4
Fine silt	1.4
Very fine silt	0.6
Clay	2.2

Table S2. Element composition of dry sediment determined by inductively coupled plasma mass spectrometry (ICP-MS).

Sediment layer	Fe (mg/kg)	Mn (mg/kg)	P (mg/kg)
0-2cm	7014	187	198
2-4cm	7462	154	162
4-6 cm	6393	124	134
6-8 cm	6781	121	125
8-10 cm	7850	170	128
10-12 cm	7840	167	141

Table S3. Metrics on the metagenomic raw sequence reads and assembled contigs obtained from Cock Soda Lake in 2016. The raw sequence reads were deposited to the NCBI Sequence Read Archive (SRA, Accession Number SRP144042).

**Metagenome ID**	**CS-Br16**	**CS-Sed16-2cmA**	**CS-Sed16-2cmB**	**CS-Sed16-5cm**
**Sample type**	brine	Top 2cm sediment	Top 2cm sediment	Top 5cm sediment
**Number of read pairs**	321209669	320027988	314554411	333806161
**Number of assembled contigs ≥1 kb**	380600	1002855	1046590	998484
**Average contig length (bp)**	2412	2560	2537	2578
**Maximum contig length (bp)**	289008	267788	248036	338372
**Total assembly length (bp)**	917990274	2567058321	2654929585	2573987824
**N50 (bp)**	2720	3018	2966	3048
**Number of contigs ≥2.5 kb**	88700	263745	268963	259716
**Number of contigs ≥5kb**	28152	87605	88336	86835

Table S4. Metrics on the metatranscriptomic raw sequence reads and assembled transcripts obtained from the top 2cm sediment layer from Cock Soda Lake in 2016. The raw sequence reads were deposited to the NCBI Sequence Read Archive (SRA, Accession Number SRP144042).

**Metatranscriptome ID**	**CS-Sed16-2cmA-RNA**
**Number of read pairs**	348450220
**% of read pairs rRNA (SortMeRNA)**	42 %
**% of read pairs used for assembly (non rRNA)**	58 %
**Number of assembled contigs ≥200 b**	1056776
**Average contig length (bp)**	719
**Maximum contig length (bp)**	43306
**Total assembly length (bp)**	760312569
**N50 (bp)**	833
