# Supplementary material for: Metagenomes and metatranscriptomes shed new light on the microbial-mediated sulfur cycle in a Siberian soda lake
Source: BMC Biol. 2019 Aug 22;17:69. doi: 10.1186/s12915-019-0688-7 (PMC6704655; doi:10.1186/s12915-019-0688-7)
Supplement: Supplementary file 5 — Figure S3. Relative abundance of top 50 abundant genera in the top 4 -cm sediment layer of Cock Soda Lake identified by amplicon sequencing of 16S rRNA genes and transcripts. (PDF 112 kb) [file 12915_2019_688_MOESM5_ESM.pdf]

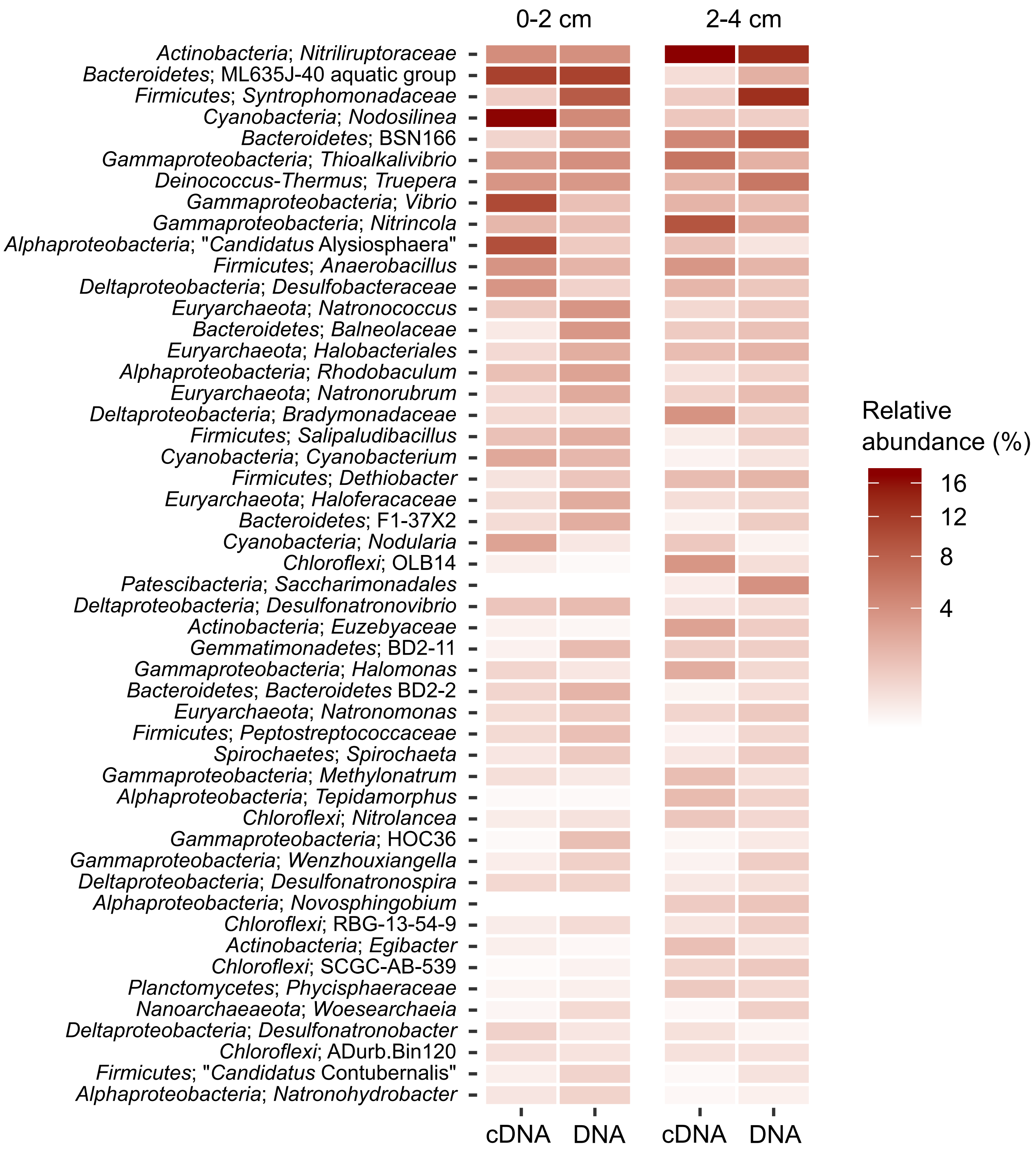

0-2 cm
2-4 cm
Actinobacteria; Nitriliruptoraceae
Bacteroidetes; ML635J-40 aquatic group
Firmicutes; Syntrophomonadaceae
Cyanobacteria; Nodosilinea
Bacteroidetes; BSN166
Gammaproteobacteria; Thioalkalivibrio
Deinococcus-Thermus; Truepera
Gammaproteobacteria; Vibrio
Gammaproteobacteria; Nitrincola
Alphaproteobacteria; "Candidatus Alysiosphaera"
Firmicutes; Anaerobacillus
Deltaproteobacteria; Desulfobacteraceae
Euryarchaeota; Natronococcus
Bacteroidetes; Balneolaceae
Euryarchaeota; Halobacteriales
Alphaproteobacteria; Rhodobaculum
Euryarchaeota; Natronorubrum
Deltaproteobacteria; Bradymonadaceae
Firmicutes; Salipaludibacillus
Cyanobacteria; Cyanobacterium
Firmicutes; Dethiobacter
Euryarchaeota; Haloferacaceae
Bacteroidetes; F1-37X2
Cyanobacteria; Nodularia
Chloroflexi; OLB14
Patescibacteria; Saccharimonadales
Deltaproteobacteria; Desulfonatronovibrio
Actinobacteria; Euzebyaceae
Gemmatimonadetes; BD2-11
Gammaproteobacteria; Halomonas
Bacteroidetes; Bacteroidetes BD2-2
Euryarchaeota; Natronomonas
Firmicutes; Peptostreptococcaceae
Spirochaetes; Spirochaeta
Gammaproteobacteria; Methylonatrum
Alphaproteobacteria; Tepidamorphus
Chloroflexi; Nitrolancea
Gammaproteobacteria; HOC36
Gammaproteobacteria; Wenzhouxiangella
Deltaproteobacteria; Desulfonatronospira
Alphaproteobacteria; Novosphingobium
Chloroflexi; RBG-13-54-9
Actinobacteria; Egibacter
Chloroflexi; SCGC-AB-539
Planctomycetes; Phycisphaeraceae
Nanoarchaeaeota; Woesearchaeia
Deltaproteobacteria; Desulfonatronobacter
Chloroflexi; ADurb.Bin120
Firmicutes; "Candidatus Contubernalis"
Alphaproteobacteria; Natronohydrobacter
cDNA
DNA
cDNA
DNA
Relative abundance (%)
16
12
8
4

Supplementary Figure 3. Relative abundance of top 50 abundant genera in the top 4 cm sediment layer of Cock Soda lake identified by amplicon sequencing of 16S rRNA genes and transcripts. Two different sediment layers were sampled (“0-2 cm” or “2-4 cm”) for parallel DNA and RNA extraction to target 16S rRNA genes (“DNA”) or 16S rRNA gene transcripts (“cDNA”), respectively. Each column in the heat map represents the average of three or four biological replicates. The minimum relative abundance of an OTU shown is 0.01% (white).

In each layer, DNA and RNA profiles varied substantially between one another. The most abundant transcripts in the top 2 cm layer were assigned to the genera *Nodosilinea* (filamentous benthic cyanobacterium) and an unknown genus within the ML635J-40 aquatic group (*Bacteroidetes*). In the 2-4 cm layer of sediments, the most abundant transcripts originated from members of the *Nitriliruptoraceae* and *Nitrincola*. Overall, the relative abundance of transcripts with phylogenetic affinity to sulfidogenic genera was higher in the top layer with the exception of *Dethiobacter*, from which transcripts were more abundant in the second layer. The relative abundance of transcripts from SOB was higher in the second layer compared to the top layer.
