# Supplementary material for: Metagenomes and metatranscriptomes shed new light on the microbial-mediated sulfur cycle in a Siberian soda lake
Source: BMC Biol. 2019 Aug 22;17:69. doi: 10.1186/s12915-019-0688-7 (PMC6704655; doi:10.1186/s12915-019-0688-7)
Supplement: Supplementary file 8 — Figure S4. Rfam family assignment of metatranscriptomic reads originating from non-coding RNA (ncRNA). Only the percentage of reads (relative to the total of 136474149 reads assigned as ncRNA) for the top 10 abundant Rfam families is shown. OLE RNAs are widespread among thermophilic Firmicutes, including sulfidogens from the genera Desulfotomaculum, but their exact function remains unknown [99]. (PDF 88 kb) [file 12915_2019_688_MOESM8_ESM.pdf]

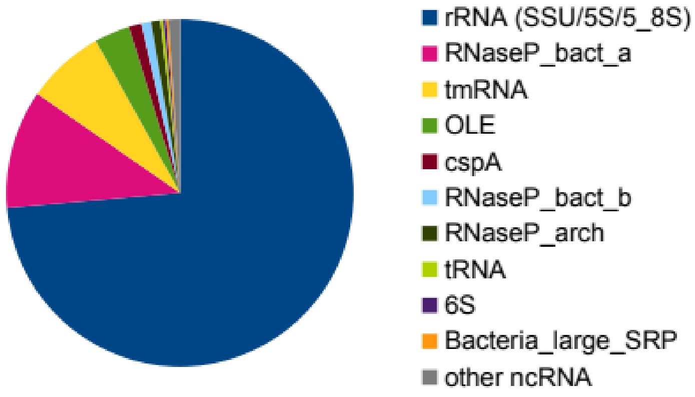

Figure S4 Rfam family assignment of metatranscriptomic reads originating from non-coding RNA (ncRNA). Only the percentage of reads (relative to the total of 136474149 reads assigned as ncRNA) for the top 10 abundant Rfam families is shown. OLE RNAs are widespread among thermophilic *Firmicutes*, including sulfidogens from the genera *Desulfotomaculum*, but their exact function remains unknown [99].
