# Supplementary material for: Metagenomes and metatranscriptomes shed new light on the microbial-mediated sulfur cycle in a Siberian soda lake
Source: BMC Biol. 2019 Aug 22;17:69. doi: 10.1186/s12915-019-0688-7 (PMC6704655; doi:10.1186/s12915-019-0688-7)
Supplement: Supplementary file 9 — Figure S5. Functional classification of the KEGG-annotated transcripts. (PDF 89 kb) [file 12915_2019_688_MOESM9_ESM.pdf]

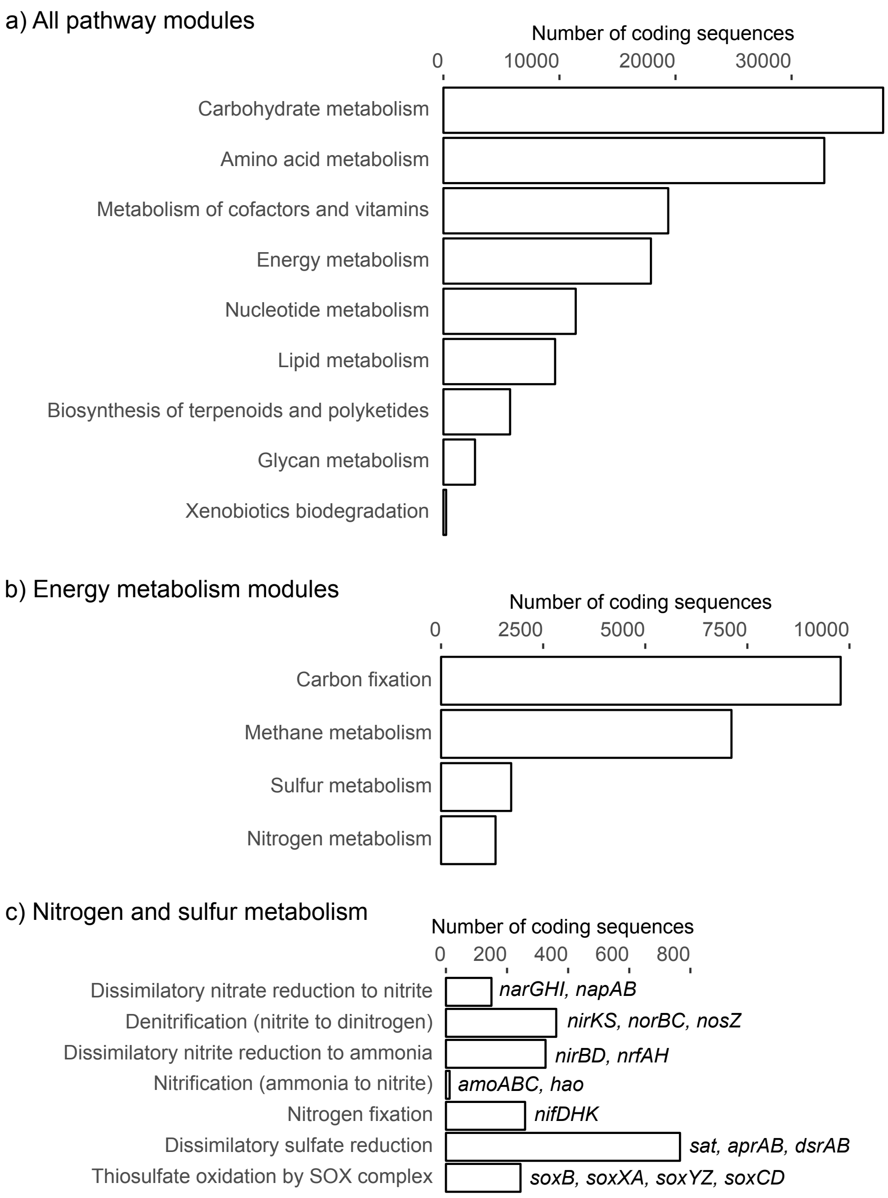

Supplementary Figure 5. Functional classification of the KEGG-annotated transcripts. In total 582,671 K numbers were assigned to 1,419,467 coding sequences (cds) on the metatranscriptomic contigs. a) Classification of annotated cds to the pathway modules within KEGG mapper. b) Sub-classification of annotated cds within “Energy metabolism”. c) Coding sequences annotated as transcripts of selected marker genes in pathways within the “Nitrogen metabolism” and “Sulfur metabolism”.
