# Supplementary material for: Metagenomes and metatranscriptomes shed new light on the microbial-mediated sulfur cycle in a Siberian soda lake
Source: BMC Biol. 2019 Aug 22;17:69. doi: 10.1186/s12915-019-0688-7 (PMC6704655; doi:10.1186/s12915-019-0688-7)
Supplement: Supplementary file 11 — Figure S6. Maximum likelihood tree showing the phylogeny of the large subunit of RubisCo found in the MAG of the putative photoautotrophic Gemmatimonadetes bacterium. (PDF 154 kb) [file 12915_2019_688_MOESM11_ESM.pdf]

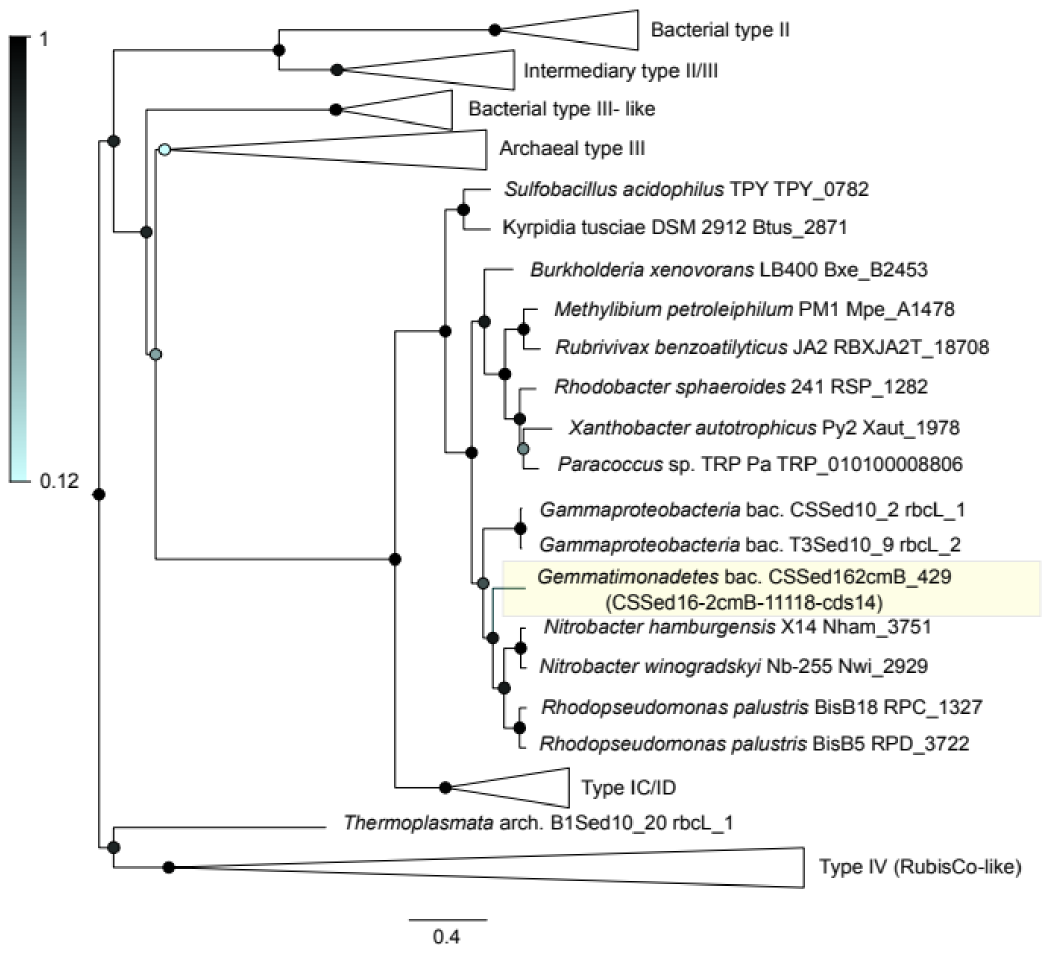

Supplementary Figure 6. Maximum likelihood tree showing the phylogeny of the large subunit of RubisCo found in the MAG of the putative photoautotrophic *Gemmatimonadetes* bacterium. References were obtained from [35]. All types except for type IA/IB are collapsed and the branch of the *Gemmatimonadetes* MAG is highlighted. Colored circles at the nodes represent the bootstraps values and the scale bare indicates the amino acid substitutions per site.
