# Supplementary material for: Metagenomes and metatranscriptomes shed new light on the microbial-mediated sulfur cycle in a Siberian soda lake
Source: BMC Biol. 2019 Aug 22;17:69. doi: 10.1186/s12915-019-0688-7 (PMC6704655; doi:10.1186/s12915-019-0688-7)
Supplement: Supplementary file 12 — FigureS7. Agarose gels showing the integrity of the total RNA used for metatranscriptomics sequencing (top 0–2-cm sediment layer) and amplicon sequencing of 16S rRNA gene transcripts (top 0–2-cm and 2–4 -cm sediment layers). (PDF 522 kb) [file 12915_2019_688_MOESM12_ESM.pdf]

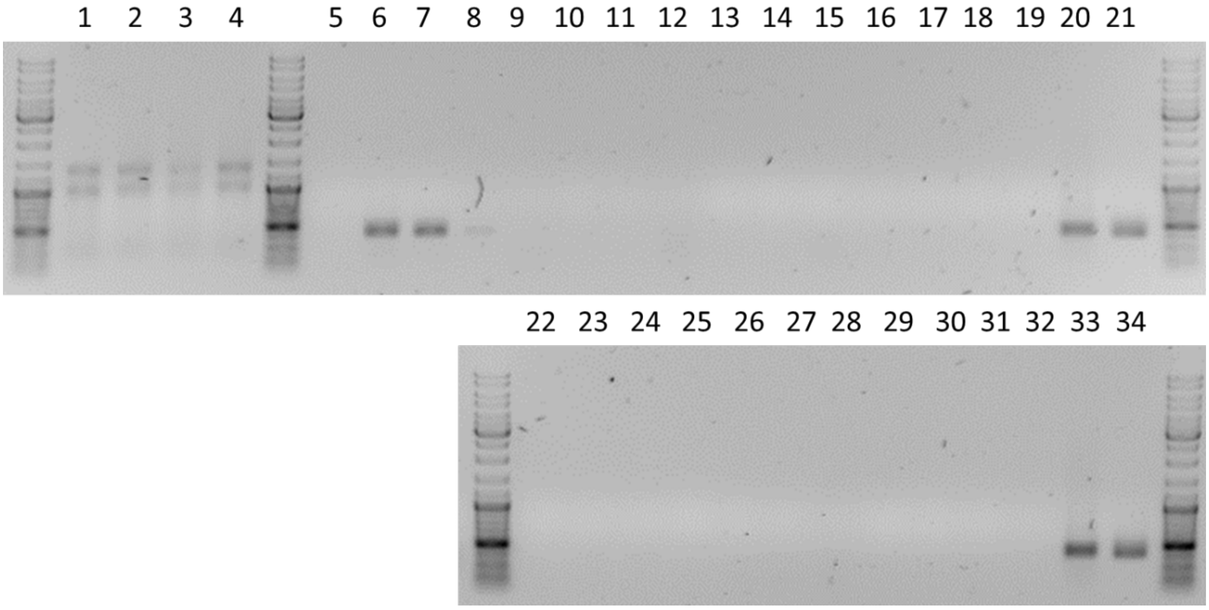

Supplementary Figure 7. Agarose gels showing the integrity of the total RNA used for metatranscriptomics sequencing (top 0-2cm sediment layer) and amplicon sequencing of 16S rRNA gene transcripts (top 0-2cm and 2-4cm sediment layers). Lane 1: Total RNA (~160ng) isolated from the top 0-2cm sediment layer, **Lane2-4:** DNase I treated RNA (~200ng). A PCR was performed on all RNA to check for residual DNA contamination (targeting 16S rRNA genes) and 25% of all PCR products were placed on the gel. For all template RNA that was sufficiently available, dilution series were included to rule out PCR inhibition, **Lane 6-8/23-25:** PCR products of untreated template RNA from 0-2cm/2-4cm sediment layer (~100-20-1 ng/30-15-1 ng template), **Lane 9-16:** PCR products of DNase I treated RNA from 0-2cm sediment layer (triplicates, ~100-20-1 ng template), **Lane 26-27:** PCR products of DNase I treated RNA from 2-4cm sediment layer (duplicates, ~15 ng template), **Lane 28-30**: PCR products of DNase I treated RNA from 2-4cm sediment layer (~30-15-1 ng template), **Lane 20/33**: positive control (DNA isolated from 0-2cm sediment layer), **Lane 21/34:** positive control (DNA isolated from 0-5cm sediment layer Cock soda lake, sampled in 2015), **Lane 18,31**: negative control (DNase I treated), **Lane 5, 19, 22, 32:** negative control (PCR-grade water). Gene rulers have a total length of 10 kb.
